# Particle-Scale Understanding of Arsenic Interactions with Sulfidized Nanoscale Zerovalent Iron and Their Impacts on Dehalogenation Reactivity

**DOI:** 10.1021/acs.est.3c08635

**Published:** 2023-12-13

**Authors:** Jiang Xu, Chaohuang Chen, Xiaohong Hu, Du Chen, Garret Bland, Jonas Wielinski, Ralf Kaegi, Daohui Lin, Gregory V. Lowry

**Affiliations:** † Zhejiang Provincial Key Laboratory of Organic Pollution Process and Control, Department of Environmental Science, Zhejiang University, Hangzhou 310058, China; ‡ Department of Civil and Environmental Engineering, Carnegie Mellon University, Pittsburgh, Pennsylvania 15213, United States; § Eawag, Swiss Federal Institute of Aquatic Science and Technology, Überlandstrasse 133, Dübendorf 8600, Switzerland

**Keywords:** arsenic remediation, antibiotic degradation, defluorination, groundwater remediation, environmental nanotechnology

## Abstract

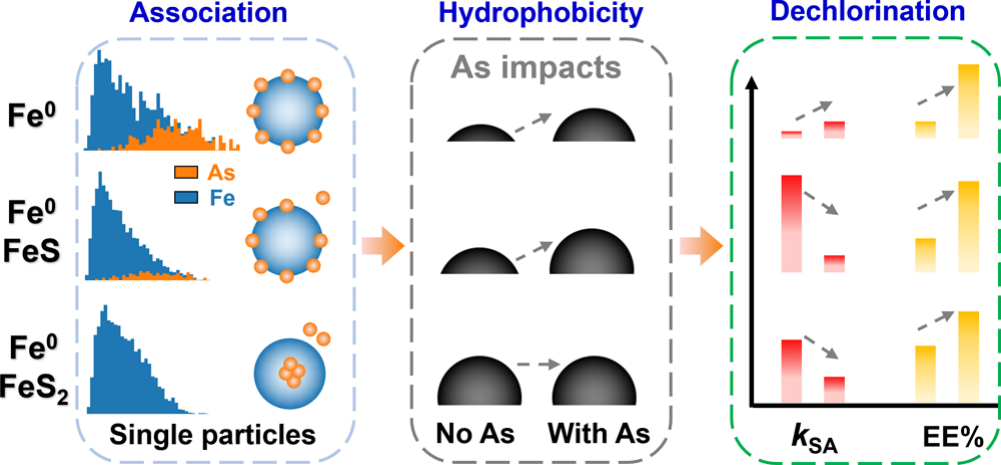

Co-occurrence of organic contaminants and arsenic oxoanions occurs often at polluted groundwater sites, but the effect of arsenite on the reactivity of sulfidized nanoscale zerovalent iron (SNZVI) used to remediate groundwater has not been evaluated. Here, we study the interaction of arsenite [As(III)] with SNZVI at the individual-particle scale to better understand the impacts on the SNZVI properties and reactivity. Surface and intraparticle accumulation of As was observed on hydrophilic FeS–Fe^0^ and hydrophobic FeS_2_–Fe^0^ particles, respectively. X-ray absorption spectroscopy indicated the presence of realgar-like As–S and elemental As^0^ species at low and high As/Fe concentration ratios, respectively. Single-particle inductively coupled plasma time-of-flight mass spectrometry analysis identified As-containing particles both with and without Fe. The probability of finding As-containing particles without Fe increased with the S-induced hydrophobicity of SNZVI. The interactions of SNZVI materials with coexisting arsenite inhibited their reactivity with water (∼5.8–230.7-fold), trichloroethylene (∼3.6–67.5-fold), and florfenicol (∼1.1–5.9-fold). However, the overall selectivity toward trichloroethylene and florfenicol relative to water was improved (up to 9.0-fold) because the surface-associated As increased the SNZVI hydrophobicity. These results indicate that reactions of SNZVI with arsenite can remove As from groundwater and improve the properties of SNZVI for dehalogenation selectivity.

## Abstract 2

Sulfidation of Fe^0^ can alter its interaction with arsenic, affecting the arsenic distribution on the particles, surface properties, and dehalogenation reactivity and selectivity.

## 1 Introduction

Groundwater is a vital freshwater resource^1^ but is susceptible to contamination from both natural and industrial sources. Arsenic (As), chlorinated solvents, and antibiotics are among the most prevalent groundwater contaminants worldwide.^2−6^ Only in rare cases are chlorinated solvents such as trichloroethene (TCE), the single contaminant of concern at a contaminated site. Arsenic is also commonly present at TCE-contaminated National Priorities List sites (U.S.).^7,8^ Antibiotics like florfenicol (FF, one of the top five antibiotics used in China, ∼6370 tons in 2013) used in animal agriculture^9^ can also co-occur with As used as an animal feed additive.^10^ Both As and FF are contained in feedlot wastewaters and potentially reach local aquifers.^11,12^ Therefore, there is an increasing need for new materials to remediate aquifers polluted with As and organic contaminants.

Nanoscale zerovalent iron (NZVI) and sulfidized nanoscale zerovalent iron (SNZVI) are used as in situ groundwater remediation agents.^13−17^ NZVI and SNZVI have the ability to reduce and sequester both arsenite [As(III)] and arsenate [As(V)]^18−22^ and can dehalogenate TCE and FF in groundwater.^23,24^ NZVI also readily reduces water, forming an iron (hydr)oxide passivation layer that can adsorb arsenate,^25^ but this passivation layer also lowers the reactivity and selectivity of NZVI toward target organic contaminants.^26,27^ The incorporation of sulfur (S) in NZVI to form SNZVI creates more and different reactive sites, increases the hydrophobicity of the particle surface, and facilitates the electron transfer toward target contaminants.^28−34^ At the same time, S-induced hydrophobicity lowers the reactivity with water, making SNZVI more selective for groundwater contaminants compared to NZVI.^23,35,36^ The amount and speciation of the sulfur in the SNZVI particles affect its properties and reactivity,^30^ but it is unclear if the sulfur amount and speciation could change the distribution and speciation of As on SNZVI particles.

There is limited mechanistic information about the chemical association of arsenic with SNZVI and its impact on SNZVI properties and reactivity. The reduction rate of arsenite by SNZVI is rapid (typically tens of minutes)^22^ compared with dehalogenation of TCE and FF by SNZVI (typically hours to days),^24,37^ so arsenite will react with SNZVI and potentially affect the SNZVI physicochemical properties and reactivity with organic compounds. Both As(III) and As(V) species have been reported to occur on the SNZVI surface,^22^ but the distribution of As species on and within the SNZVI core is uncertain, and the potential to form separate As-bearing particles (without associated Fe) is unknown. As may potentially be reduced to metallic As^0^ because the standard reduction potential of the Fe^2+^/Fe^0^ (−0.44 V) couple is lower than that of the AsO_2_^–^/As_(s)_ (0.24 V) couple. Standard X-ray techniques [e.g., XPS, X-ray diffraction, and X-ray absorption spectroscopy (XAS)] provide only average As speciation of the dried SNZVI particles and do not provide any *individual particle* information. In contrast, single particle inductively coupled plasma time-of-flight mass spectrometer (spICP-TOF-MS) can determine the formation of separate As-bearing particles because it characterizes the full elemental composition of *individual nanoparticles* (NPs).^38−40^ Regular ICP–MS measures either As or Fe in solution, so the coassociations of As and Fe in the same particle cannot be determined. Characterizing thousands of individual particles by spICP-TOF-MS may help explain the observed bulk reactivity between arsenite and SNZVI.

The interaction of arsenic with SNZVI and its influence on SNZVI reactivity and selectivity toward organic compounds is currently unknown. Moreover, the influence of Fe and S speciation in the SNZVI particles on the reactivity with arsenite is unknown. As(III) was selected as the representative arsenic species because the difference between As(III) and As(V) removals by Fe^0^-based materials has been previously reported,^18−22^ and because As(III) is more toxic than As(V) and the most likely arsenic species to be present under reducing conditions (e.g., anoxic groundwater) expected in the presence of SNZVI. Therefore, in this work, we exposed NZVI or SNZVI with different sulfur contents (low, medium, and high) to arsenite at low and high As/Fe ratios, to assess if SNZVI could be a better version than NZVI for mixed contamination. Two probes (i.e., TCE and FF) were used to better understand how the interactions between arsenite and the materials may affect their reactivity and selectivity toward dehalogenation. Reaction products were investigated using transmission electron microscopy-coupled energy-dispersive X-ray spectroscopy (TEM–EDX), spICP-TOF-MS, and XAS. Results revealed the arsenic distribution and its association with other elements and its impacts on the SNZVI materials’ properties and reactivity with water and the target organic contaminants TCE and FF.

## 2 Experimental Section

### 2.1 Materials

FeCl_3_ (97%) was obtained from Sigma-Aldrich. NaBH_4_ (98%), NaAsO_2_ (>90%), and TCE (≥99.5%) were purchased from Fisher Scientific. Na_2_S_2_O_4_ (≥88%) was obtained from Sinopharm Chemical Reagent Co., Ltd., China. FF (>99%) and its dechlorinated products (deschloro FF and dideschloro FF) were obtained from Shanghai Ruichu Biotech Co., Ltd. and Absin Bioscience Inc., respectively.

Water contact angle (WCA) measurements were used to assess the hydrophobicity of materials. NZVI (WCA = 18 ± 4°), [S/Fe]_particle_ = 0.010 SNZVI (Fe^0^/FeS, WCA = 48 ± 4°), [S/Fe]_particle_ = 0.049 SNZVI (Fe^0^/FeS_2_, WCA = 112 ± 2°), and [S/Fe]_particle_ = 0.099 SNZVI (Fe^0^/FeS/FeS_2_, WCA = 78 ± 3°) were synthesized and characterized for hydrophobicity according to a previously reported one-step synthesis method,^30^ where a Na_2_S_2_O_4_ and NaBH_4_ solution was dropwise added into a Fe^3+^ solution.

### 2.2 Batch Experiments

Batch reactivity experiments were conducted in 160 mL capped serum bottles containing 100 mL of solution and 60 mL of headspace. The removal kinetics of arsenite (NaAsO_2_) was determined over a 180 min reaction. The adsorption isotherm of arsenite by SNZVI was determined at different concentrations of arsenite (1–200 mg L^–1^ As) after a 24 h reaction. 10 and 100 μL of 1 g L^–1^ As(III) stock solution was spiked into the 100 mL of 1 g L^–1^ materials that represent “As-unsaturated_low_” (100 μg L^–1^) and “As-unsaturated_high_” (1 mg L^–1^) scenarios, respectively. 1 mL of 10 g L^–1^ As(III) stock solution was gradually spiked into the 99 mL of 1 g L^–1^ materials to simulate the scenario that the materials were loaded with high amount of As or saturated with As, i.e., “As-unsaturated” scenario. The “unsaturated” condition refers to a case where all of the added As(III) could be theoretically removed by the particles and therefore the surface is unsaturated with respect to As association. The “saturated” condition refers to a case of high As/Fe ratio where As(III) is present in excess or the iron material is limited, thereby saturating the surface of the NZVI or SNZVI surfaces.

The reactivity of each As-reacted NZVI and SNZVI material with water, TCE solution (70 μM), or FF solution (28 μM) was measured to determine the potential impacts of the associated arsenic species on the reactivity and selectivity of the NZVI and SNZVI materials toward target halogenated compounds. Considering the much faster removal rates of arsenite (minutes to hours) by SNZVI as compared to TCE (days) and FF (hours to days), the impact of coexisting arsenite on the TCE or FF removal is supposed to be more remarkable than the converse scenario for an arsenite-TCE or arsenite-FF cocontaminated groundwater. Hence, TCE or FF was added to the arsenite-reacted SNZVI suspension (24 h to reach equilibrium), the materials of which were then characterized by multiple techniques. In all experiments, deoxygenated deionized (DI) water (pH = 5.5) after 1 h N_2_ purging was used, the bottles were capped by Teflon Mininert valves, and the initial pH was 5.5 and rotated on an end-overend rotator at 30 rpm at 25 ± 2 °C.

The arsenite reactivity and its impacts on dehalogenation of SNZVI were also assessed using real groundwater sampled from Hangzhou, China. Detailed information on the physicochemical parameters of the groundwater was reported in our previous study (Table S1).^41^ Environmentally relevant concentrations of As(III) were used, i.e., 1 mg L^–1^ As(III) for TEM–EDX analysis to allow the detection of As and 100 μg L^–1^ As(III) for evaluating its impacts on the dehalogenation reactivity and selectivity of SNZVI. The concentrations of TCE/FF and other reaction conditions were the same as the above batch experiments in DI water.

### 2.3 Analytical Methods

Gas chromatography with thermal conductivity (GC-TCD, Agilent 6850) and flame ionization detectors (GC-FID, HP 6890) were used to quantify H_2_ in the headspace and the TCE and main reduction products (C_2_H_2_, C_2_H_4_, and C_2_H_6_), respectively. The electron efficiency (defined as the fraction of electrons donated by Fe^0^ that are used to reduce TCE or FF) was calculated when the majority (>95%) of TCE or FF was removed or at the end of reaction (8 and 15 days for TCE and FF, respectively). Detailed analysis methods, calculations, and materials characterizations (e.g., surface area, TEM–EDX, and WCA) were conducted, as previously reported.^30,42^

The As-unsaturated_high_ and As-saturated materials with sufficiently high As concentrations were analyzed by XAS, spICP-TOF-MS, and TEM–EDX to determine the As speciation, the chemical compositions of individual particles, and the spatial distribution of selected elements within individual particles.

#### 2.3.1 XAS Analysis

Vacuum-dried As(III)-treated SNZVI samples (after 24 h of reaction) were ground in an inert environment. A known mass of the sample was diluted in a cellulose matrix, pressed into pellets, and sealed with a Kapton tape. The models and samples were shipped to Advanced Photon Source (APS, Lemont, IL, USA). The As K-edge (11,866 eV) XAS data were collected on beamline 20BM at room temperature. A Si 111 monochromator was used to focus the energy beam. The pre-edge was measured from 11,667 to 11,847 eV in 10 eV intervals, the X-ray absorption near-edge structure (XANES) spectra were measured from 11,847 to 11,917 eV in 0.40 eV intervals, and the EXAFS spectra were measured from 11,917 to 12,722 eV (15 wavenumber) in 0.05 wavenumber intervals. A gold standard was measured for each sample to correct for any eV shift. Each set of sample spectra were then merged and analyzed for linear combination fitting (LCF) (1.5 to 10.0 Å^–1^ for EXAFS, −10 to 22 eV for XANES) by ATHENA software from the Demeter XAS analysis package.^43^

#### 2.3.2 spICP-TOF-MS Analysis

As(III)-treated NZVI or SNZVI suspensions [1 g L^–1^ materials with As(III) after 24 h reaction] were diluted by 10^5^ times and immediately analyzed by spICP-TOF-MS 1R model from TOFWerk (Thun, Switzerland). A full description of the instrument and the data analysis are described in previous studies. The instrument is operated in the vented mode, and As and all Fe isotopes were measured. S was not measured due to low isotope abundance or external isotope interferences (e.g., O_2_ and ^32^S). Dissolved Fe and As calibrations between 0 to 5 ppb were used to quantify the particles in each measurement. A 40 nm Au NP standard solution (nanoComposix) was used to determine the transport efficiency.^44^ The nebulizer gas flow rate, nebulizer liquid flow rate, attenuated masses, TOF extraction frequency, cooling gas flow rate, and plasma power are reported in another recent study.^40^ The full description of identifying particle events from the baseline signal is described elsewhere.^45^ An integration time of 0.002 s was used to identify individual particle events.^45^ To account for large particle sizes, up to three consecutive events were merged to one. All spICP-TOFMS data were processed with TOFPilot software. The format of the single particle data was given in time series for all of the measured isotopes in csv format. Post processing analysis was performed in Python.

## 3 Results and Discussion

### 3.1 Arsenite Reactivity and Elemental Distribution of As-Reacted SNZVI

The As removal kinetics and As adsorption capacity of SNZVI were investigated first (Figure S1 and Table S2). A second-order kinetic model fit the As adsorption data for all of the materials, while sulfur could turn the adsorption isotherm of As from a Langmuir model to a Freundlich model. The relevant discussion is provided in Text S1. All of the added As was removed (Figure S1) and evenly distributed over the NZVI and SNZVI particles at a low As/Fe ratio (Figures S2–S4). It is difficult to distinguish whether As was distributed on the surface or inside the particles, or both, due to the relatively low concentration of As. These As distributions could affect the reactivity and selectivity of SNZVI materials with coexisting contaminants, as discussed later. A few S-rich clusters were present on the SNZVI (Figure S3f-0.010 SNZVI and Figure S4f-0.049 SNZVI), but As was not specifically enriched in these spots, suggesting a poor correlation between As and S despite the potential to form As–S species like realgar.^46^

**Figure 1:**
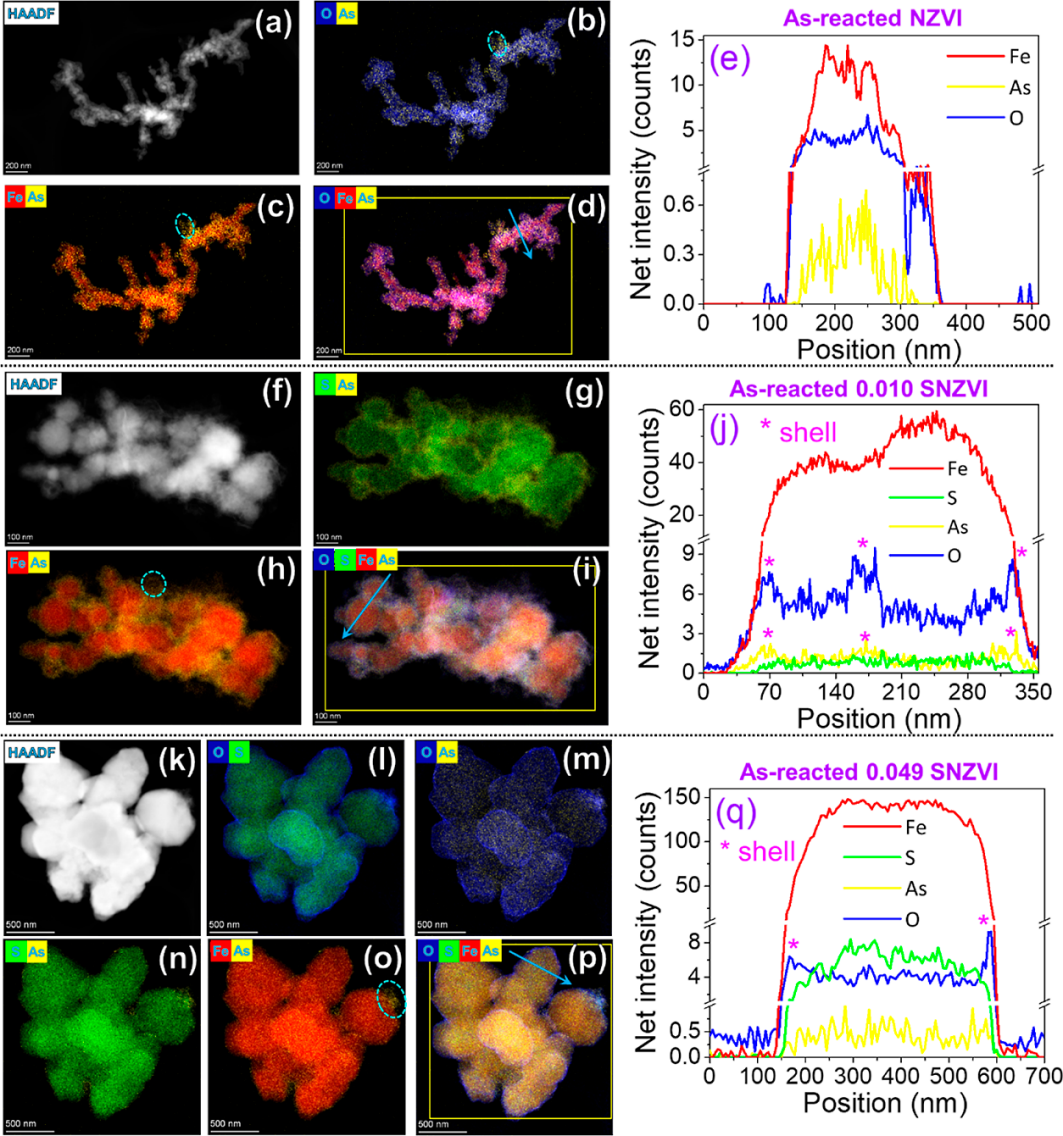
High-angle annular dark-field (HAADF) images and elemental distribution of “As-saturated” (a–e) NZVI, (f–j) 0.010 SNZVI (Fe^0^/FeS), and (k–q) 0.049 SNZVI (Fe^0^/FeS_2_).

The presence of sulfur altered the distributions of As in the SNZVI compared to NZVI at a high As/Fe ratio (Figures 1 and S5–S7). For As-saturated NZVI (no sulfur), the O was mainly present as an iron (hydr)oxide shell with a high affinity for As (Figure S5d).^47,48^ The As-saturated 0.010 SNZVI (Fe^0^/FeS) particles also revealed that the As shell (Figure 1h,j) was better correlated with the O atoms (Figure S6e,l) than the S atoms (Figure S6m). The S was evenly distributed throughout the particles rather than being present as a shell (Figure S6f,g), suggesting that the As on the particle surfaces was predominantly associated with iron (hydr)oxides. When the amount of sulfur incorporated into the particles is increased (0.049 SNZVI particles), As becomes more evenly distributed throughout the particles rather than being concentrated on the O-bearing shell (Figure 1k–q). Reasons for this are unclear. One possible reason could be that the hydrophobic SNZVI material was less reactive with water to form Fe-oxyhydroxides, and a relatively thin Fe-oxyhydroxide shell might favor the As impregnation into the particles. The relatively high As concentration at a high As/Fe ratio saturated the Fe-oxyhydroxide shell, allowing additional As species to become associated with the sulfur in the particles. A similar phenomenon was reported for U(VI)-treated NZVI.^49^ In addition, a few As-rich clusters were observed near the shell of As-saturated NZVI and SNZVI materials (cyan circles in Figure 1c,h,o), suggesting the potential formation of other As species (e.g., As^0^ cluster) as discussed later.

### 3.2 As Speciation of the As-Treated SNZVI Materials

**Figure 2:**
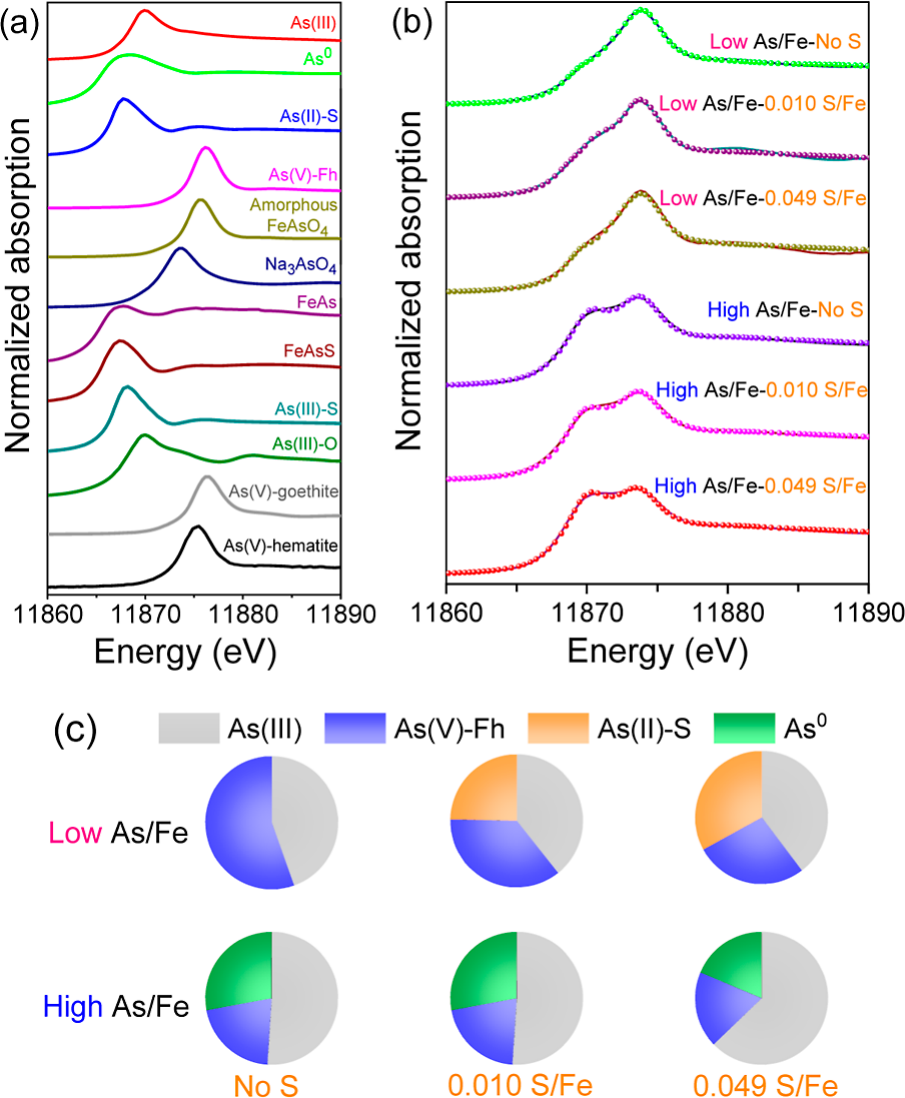
Speciation of As after 24 h reaction with SNZVI with different S amounts in As-unsaturated (i.e., low As/Fe ratio) and As-saturated (i.e., high As/Fe ratio) scenarios. (a) XANES spectra of As reference compounds, including *sodium arsenite* [As(III), NaAsO_2_] and *sodium arsenate* (Na_3_AsO_4_), *As-adsorbed iron (hydr)oxides* (ferrihydrite, goethite, and hematite), *arsenic sulfides*-orpiment [As(III)-S] and realgar [As(II)-S], *arsenic oxide* [As(III)-O], *elemental arsenic* (As^0^), *iron arsenide* (FeAs), *arsenopyrite* (FeAsS), and *amorphous FeAsO*_*4*_. (b) Linear combination fits of XANES spectra at As K-edge and (c) their fitted results. Line denotes the sample, while spherical marker denotes the fit.

The bulk As speciation was estimated by LCF of the As K-edge XANES (Figure 2 and Table S3). All of the samples contained some fraction of As(V)-Fh species (Figure 2), indicating the partial oxidation of arsenite, consistent with a recent study on arsenite removal by SZVI.^50^ It may be an artifact of the samples being exposed to air despite taking precautions to avoid air exposure. However, it is also possible that the hydroxyl radical was responsible for the observed As oxidation. A recent study reported that hydroxyl radical can form from surface-bound Fe(II) on SZVI and H_2_O_2_, which was generated via a surface-mediated reaction between iron sulfides and water under anaerobic conditions.^51^ The As speciation was significantly affected by the As/Fe ratio used. There was formation of a realgar-like arsenic sulfide species [As(II)–S] under the As-unsaturated_high_ scenario. However, this species was not observed when the SNZVI was exposed to excess As (i.e., As-saturated scenario). Instead, a fraction of the As was reduced to elemental As^0^ that was detected in both As-NZVI and As-SNZVI particles, consistent with the As-bearing clusters observed in the TEM maps (Figure 1). Limited contributions of iron arsenide (FeAs) or arsenopyrite (FeAsS) in the LCF suggests that these two compounds were not present or that their concentration is below the detection limit of XAS. The relatively large fraction of As(III) suggests that a majority of the As is adsorbed, likely to the Fe-oxyhydroxides present on the particle surfaces. The amount of sulfur (0.010 or 0.049 [S/Fe]_particle_) and speciation of sulfur in the particles (FeS vs FeS_2_) did not significantly affect the speciation of As, most likely because of a similar affinity of FeS and FeS_2_ toward As (Figure S8).

Overall, the results in these studies indicate that the speciation of As in SNZVI exposed to arsenite will depend on the ratio of As/Fe in the system. Most natural systems would tend to have low As concentrations, suggesting that As(V)-Fh, As(II)-S, and adsorbed As(III) will be the likely species present on SNZVI. However, this could change as the material ages, and the As concentration on the particle surfaces increases. The temporal evolution of the As speciation and stability in these materials merits further investigation.

### 3.3 Arsenic Associations at the Individual Particle Scale

**Figure 3:**
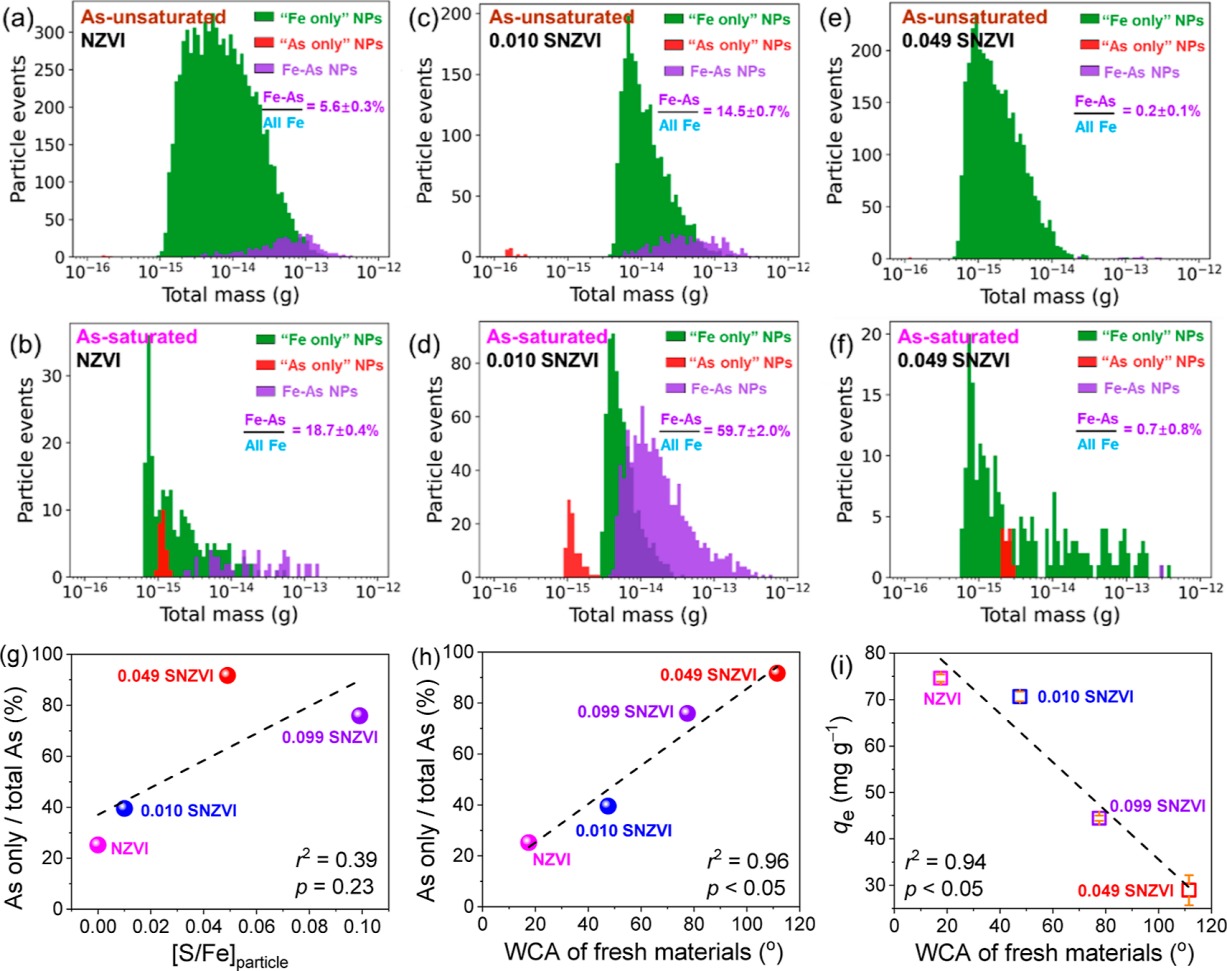
(a–f) Mass distribution of ^54^Fe and ^75^As-bearing NPs at the individual particle level for As-treated hydrophilic NZVI, hydrophilic SNZVI ([S/Fe]_particle_ = 0.010), and hydrophobic SNZVI ([S/Fe]_particle_ = 0.049) according to spICP-TOF-MS analysis (probability of ^75^As–^54^Fe bearing NPs as % of the total ^54^Fe NPs). Correlations of (g) [S/Fe]_particles_ and (h) hydrophobicity of fresh materials with the probability of “^75^As only” (as % of the total ^75^As-bearing NPs), and (i) correlation between the hydrophobicity of fresh materials and the adsorbed As amount in the As-saturated scenario.

To complement the bulk XAS analyses, the As and Fe associations of individual particles were analyzed by spICP-TOF-MS. More than 8000 Fe-bearing NPs were analyzed in the As-NZVI and As-0.010 SNZVI suspensions in the “As-unsaturated_high_” scenario (Figure 3a,c), with 5.6 and 14.5% of the total Fe NPs associated with As, respectively. The rest of Fe NPs was present as “Fe only” NPs, either present as iron (hydr)oxides or associated with too low of an As mass to be detected (detection limit, 1.70 × 10^–16^ g). More Fe NPs were associated with As (18.7 and 59.7%) in the “As-saturated” scenario (Figure 3b,d). Interestingly, ∼25 and ∼40% of As-bearing NZVI and 0.010 SNZVI were present as “As only” NPs, respectively, either associated with very limited ^54^Fe mass (below the detection limit, 2.18 × 10^–16^ g) or as As-only particles (e.g., As^0^, as shown in Figure 2 or As–S particles if any). A similar trend of an increased probability of As-associated Fe NPs in the “As-saturated” scenario was also observed for the hydrophilic 0.099 SNZVI (Figures S9 and S10).

For the hydrophobic 0.049 SNZVI, very few Fe NPs (0.2 and 0.7%) were found to be associated with As (Figures 3e,f and S9c,d). There could be As in the Fe NPs but without sufficient mass to be detected as a particle event by spICP-TOF-MS, consistent with the lowest As removal by this material (Figure S1h). A possible explanation for this finding is that As was evenly distributed over SNZVI particles, but the amount of As on individual SNZVI particles was too limited (Figure S7) to be detected by spICP-TOF-MS. Alternatively, As was primarily present as “As only” NPs [e.g., As(II)-S and As^0^ clusters in the “As-unsaturated_high_” and “As-saturated” scenarios, respectively, as suggested by the XANES analysis in Figure 2].

The probability of finding particles with As but without Fe (i.e., “As only” NPs) as % of the total As-bearing NPs was further correlated with material properties. This probability presented a weak (*r*^2^ = 0.39, *p* = 0.23) and strong (*r*^2^ = 0.96, *p* < 0.05) positive correlation with the S content and hydrophobicity of fresh materials, respectively (Figures 3g,h and S11). Although hydrophilic arsenite appeared to penetrate into the hydrophobic SNZVI particles (Figure 1m), their contact with the hydrophobic surface would be limited, thus favoring the formation of As-containing NPs without Fe, including the formation of elemental As^0^ particles, given the highly reducing environment. It is consistent with the strong negative correlation (*r*^2^ = 0.94, *p* < 0.05) between the As adsorption capacity and hydrophobicity of SNZVI (Figure 3i). This suggests that the hydrophobicity of the materials could be used as an indicator for the removal capacity of arsenite. This is likely true for other hydrophilic heavy metal ions, e.g., the removal capacity of Cr(VI) and Cd(II) by SNZVI synthesized via the same sulfidation process first decreased then increased as the S amount increased,^52−54^ consistent with the trend of SNZVI hydrophobicity.

### 3.4 As-Induced Surface Hydrophobicity due to Reaction with Arsenite

**Figure 4:**
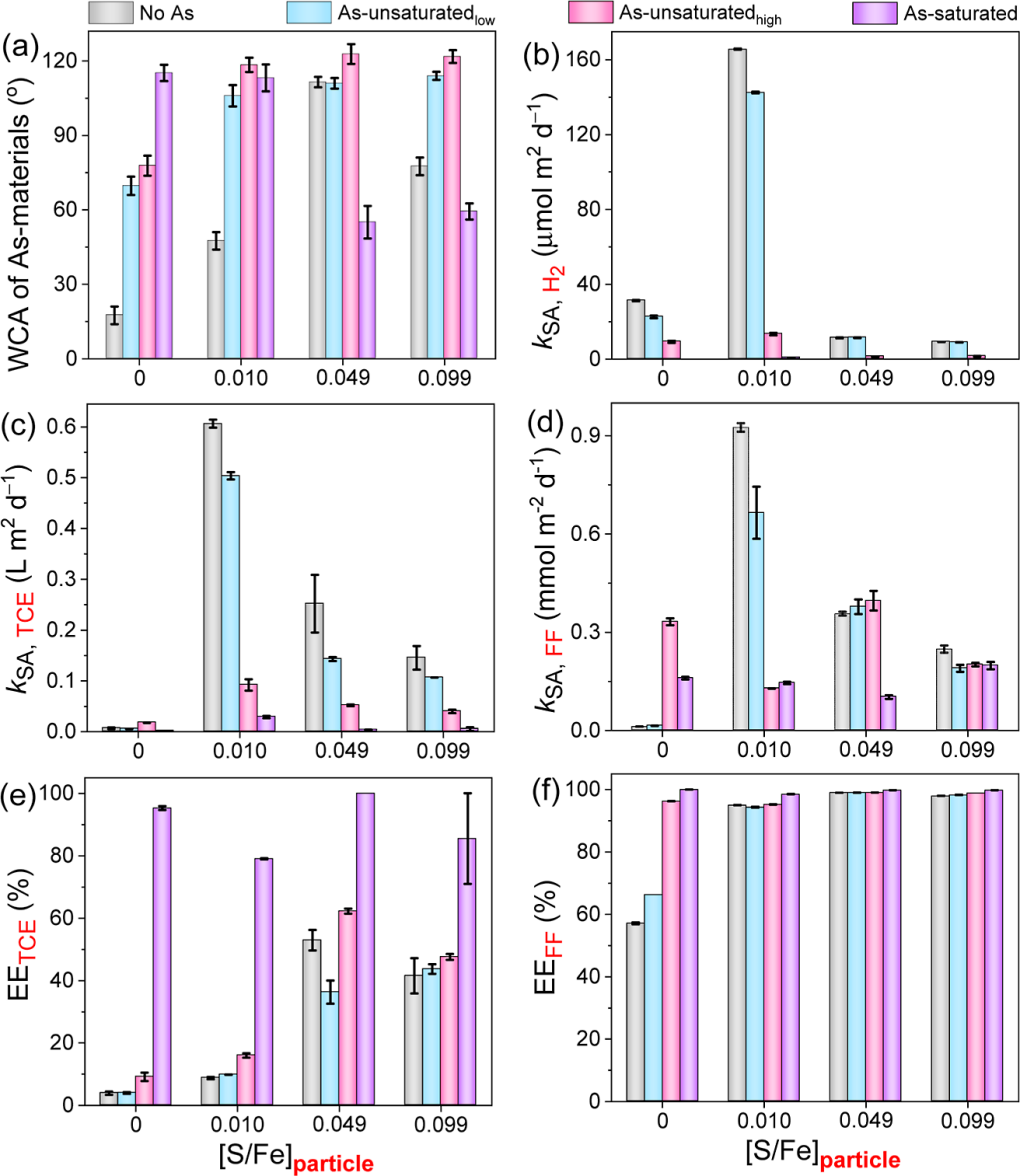
Impacts of reacted and/or adsorbed arsenite on the (a) hydrophobicity, (b) water reactivity, (c) TCE reactivity, (d) FF reactivity, electron efficiency (EE) for (e) TCE, and (f) FF degradation of SNZVI (1.0 g L^–1^ SNZVI, initial pH = 5.5, *T* = 25 ± 2 °C).

The association of As on the NZVI and SNZVI surfaces affected their hydrophobicity (Figures 4a and S12), which largely depended on the amount, speciation, and spatial distribution of As in the particles. Arsenic has a similar electronic structure to that of the nearby *p*-block elements (S, N, and P). Enhancement of the hydrophobicity of catalytic sites by these *p*-block elements has been previously reported,^55,56^ and incorporation of S, N, or P into the Fe^0^ nanoparticles can also induce hydrophobicity.^30,57,58^ This is consistent with the increased hydrophobicity of NZVI and 0.010 SNZVI materials, where most of the As was accumulated on the surface. For the already hydrophobic 0.049 and 0.099 SNZVI materials, the hydrophobicity was increased by adding a small amount of As, likely a result of the formation of arsenic minerals [e.g., realgar-like As(II)-S, as detected by XANES LCF in Figure 2] which are reported to be hydrophobic.^59^ However, a lower hydrophobicity was observed in the “As-saturated” scenarios for the 0.049 and 0.099 nm SNZVI materials. This may be because less As interacted with materials and As was more evenly distributed throughout these materials rather than that on the surface (Figure 1k–q). Note that the inevitable reaction between materials and water (despite being significantly inhibited) to form a hydrophilic Fe-oxyhydroxide shell could also decrease the hydrophobicity. Overall, these results indicated that reaction with arsenite can affect the particles’ surface hydrophobicity and can be present as individual As(II)-S or potentially As^0^ particles rather than uniformly distributed onto the NZVI or SNZVI particles. The higher particle hydrophobicity affected the particle reactivity with water and with target contaminants, as discussed below.

### 3.5 Co-Existing As Affects the Reactivity and Selectivity of SNZVI

The associated As and its induced hydrophobicity (Figure 4a) significantly suppressed the reactivity of NZVI and SNZVI with water (surface-area normalized H_2_ evolution rates, *k*_SA, H2_) in the “As-unsaturated” scenarios by 3.4-fold and 5.8–12.4-fold, respectively (Figures 4b, S13, and S14). The materials stopped reacting with water in the As-saturated scenario (i.e., no H_2_ was detected), except for the most reactive 0.010 SNZVI material (which still decreased by 230-fold).^30^ The discrepancy between the improved hydrophobicity (Figure 4a) and suppressed water reactivity (Figure 4b) across different materials suggests that the As distribution and speciation will also affect the reactive lifetime and potentially the reaction rates with target organic compounds.

The surface-area-normalized removal rates of TCE (*k*_SA, TCE_) and FF (*k*_SA, FF_) by SNZVI decreased after the reaction with arsenite (Figures 4c,d, and S15–S23). For instance, the *k*_SA, TCE_ values decreased by 6.6–20.9, 4.8–67.5, and 3.6–28.1-fold for 0.010, 0.049, and 0.099 SNZVI, respectively. The relatively large range of effect on reactivity with TCE is likely due to the range of different As species and the resulting hydrophobicity of these different starting materials. In contrast, associated As enhanced the reactivity of NZVI with TCE and FF to a maximum at 1 mg As per gram of Fe. Additional As then decreased reactivity. For both NZVI and SNZVI, the selectivity toward TCE or FF (i.e., electron efficiency) improved as the amount of associated As increased (Figure 4e,f), generally consistent with the trends in materials’ hydrophobicity (Figure 4a) and decrease in the reaction with water (Figure 4b). In addition, the maximum reactivity and selectivity of SNZVI were all better than those of NZVI under the same conditions, suggesting that SNZVI is a better material than NZVI for TCE and FF removal in the presence of arsenite.

### 3.6 Interactions and Reactivity under Real Groundwater Conditions

**Figure 5:**
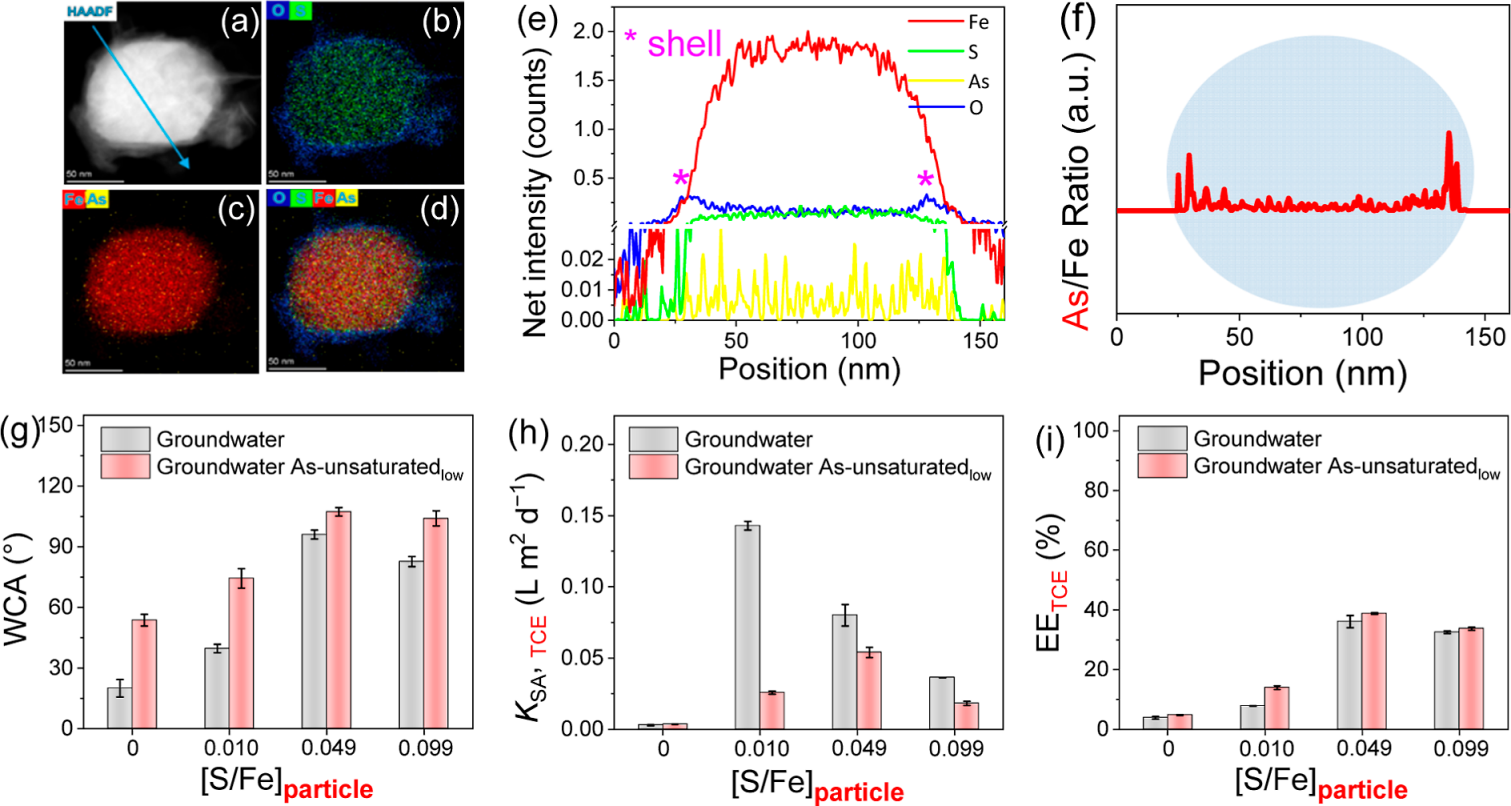
Arsenite interactions with SNZVI in real groundwater and their impacts on the TCE reactivity and selectivity. (a–f) HAADF images, elemental maps, and line–scan profile of As-reacted 0.049 SNZVI [1.0 g L^–1^ SNZVI, 1 mg L^–1^ As(III), initial pH = 5.5, *T* = 22 ± 2 °C]. (g) Hydrophobicity, (h) TCE reactivity, and (i) TCE selectivity of SNZVI with different S amounts and speciation in the presence and absence of arsenite [1.0 g L^–1^ SNZVI, 100 μg L^–1^ As(III), initial pH = 5.5, *T* = 25 ± 2 °C].

The interaction of arsenite with SNZVI and its impacts on the material properties and reactivity were tested using real groundwater. The elemental distribution maps and the ratio of As to Fe intensity over the analyzed particle indicate that arsenite reacted with the 0.049 SNZVI in real groundwater (Figures 5a–f and S24). The arsenite interaction in the real groundwater increased the hydrophobicity of all the studied materials except 0.049 SNZVI (Figures 5g and S25), inhibited their TCE reactivity (Figures 5h, S26, and S27), and slightly increased the electron efficiency toward TCE dechlorination (Figure 5i). These are consistent with the findings in DI water (Figure 4) despite the presence of dissolved ions and organic matter. The TCE reactivity of SNZVI in the real groundwater (Figure 5h) was relatively lower than that in DI water (Figure 4c), regardless of the S amount and speciation, which was likely due to the impacts of groundwater constituents, as previously reported.^60^

## 4 Environmental Implications

These results indicate that the sulfur amount and speciation in SNZVI can affect the resulting As distribution and association, which in turn affect the hydrophobicity of the resulting materials and their reactivity and selectivity with the coexisting TCE or FF. The As-induced increase of particle hydrophobicity due to reaction with arsenite lowers the reactivity with water enough that it provides high selectivity for the organic contaminants over water, thereby extending the reactive lifetime of the material. This is beneficial for in situ groundwater remediation applications as it extends barrier performance and can increase the time between injections. Even with the improvements afforded to NZVI by reaction with arsenite, the SNZVI materials still have more favorable reactivity and selectivity compared to NZVI in the presence of arsenite and TCE/FF. Better understanding of spatially resolved distribution of As species in SNZVI with varied S amounts would further advance the design of SNZVI materials since differences in speciation and distribution affect the general behavior of (S)NZVI toward coexisting contaminations. The application of SNZVI for the remediation of groundwater containing multiple inorganic and organic contaminants needs to consider their speciation and distribution in the materials.

## Supporting Information Available

Chemicals and experimental methods, XAS and spICP-TOF-MS analysis methods and data, As removal kinetics and capacity, TEM images and elemental distribution, surface area, WCA, water reactivity, TCE reactivity, and FF reactivity of the As-reacted NZVI and SNZVI (PDF)
