# Supplementary material for: Particle-Scale Understanding of Arsenic Interactions with Sulfidized Nanoscale Zerovalent Iron and Their Impacts on Dehalogenation Reactivity
Source: Environ Sci Technol. 2023 Dec 13;57(51):21917–26. doi: 10.1021/acs.est.3c08635 (PMC10753793; doi:10.1021/acs.est.3c08635)
Supplement: Supplementary file 1 — es3c08635_si_001.pdf [file es3c08635_si_001.pdf]

Supporting Information

Particle-Scale Understanding of Arsenic Interactions with Sulfidized Nanoscale Zerovalent Iron and Their Impacts on Dehalogenation Reactivity

Jiang Xu[a, *], Chaohuang Chen[a], Xiaohong Hu[a], Du Chen[a], Garret Bland[b], Jonas Wielinski[b], Ralf Kaegi[c], Daohui Lin[a], Gregory V. Lowry[b, *]

[a] Zhejiang Provincial Key Laboratory of Organic Pollution Process and Control, Department of Environmental Science, Zhejiang University, Hangzhou 310058, China

[b] Department of Civil and Environmental Engineering, Carnegie Mellon University, Pittsburgh, PA 15213, United States

[c] Eawag, Swiss Federal Institute of Aquatic Science and Technology, Überlandstrasse 133, 8600 Dübendorf, Switzerland

* Corresponding author E-mail address:

xujiang6@zju.edu.cn (J. Xu)

glowry@cmu.edu (G.V. Lowry)

Number of Pages: 33

Number of Figures: 27

Number of Tables: 3

Table of Contents

Text S1 As removal kinetics and capacity by SNZVI. **Page S3**

Figure S1 As removal kinetics and capacity by SNZVI. **Page S5**

Figure S2 HAADF and elemental maps of NZVI at low As/Fe. **Page S6**

Figure S3 HAADF and elemental maps of 0.010 SNZVI at low As/Fe. **Page S7**

Figure S4 HAADF and elemental maps of 0.049 SNZVI at low As/Fe. **Page S8**

Figure S5 HAADF and elemental maps of NZVI at high As/Fe. **Page S9**

Figure S6 HAADF and elemental maps of 0.010 SNZVI at high As/Fe. **Page S10**

Figure S7 HAADF and elemental maps of 0.049 SNZVI at high As/Fe. **Page S11**

Figure S8 q_e and k_d of As by different iron and sulfur compounds. **Page S12**

Figure S9 spICP-TOF-MS of Fe mass distribution at low As/Fe ratio. **Page S13**

Figure S10 spICP-TOF-MS of Fe mass distribution at high As/Fe ratio. **Page S14**

Figure S11 spICP-TOF-MS of As mass distribution at high As/Fe ratio. **Page S15**

Figure S12 Water contact angle measurements for As-reacted SNZVI pellets. **Page S16**

Figure S13 Impact of arsenite on the water reactivity of NZVI and SNZVI. **Page S17**

Figure S14 BET surface area of fresh and As-reacted SNZVI materials. **Page S18**

Figure S15 Impact of arsenite on the TCE removal by NZVI. **Page S19**

Figure S16 Impact of arsenite on the TCE removal by 0.010 SNZVI. **Page S20**

Figure S17 Impact of arsenite on the TCE removal by 0.049 SNZVI. **Page S21**

Figure S18 Impact of arsenite on the TCE removal by 0.099 SNZVI. **Page S22**

Figure S19 Aqueous Fe concentration after TCE reaction by SNZVI. **Page S23**

Figure S20 FF removal by NZVI or SNZVI without As. **Page S24**

Figure S21 FF removal by NZVI or SNZVI in As-unsaturated$_{low}$ scenario. **Page S25**

Figure S22 FF removal by NZVI or SNZVI in As-unsaturated$_{high}$ scenario. **Page S26**

Figure S23 FF removal by NZVI or SNZVI in As-saturated scenario. **Page S27**

Figure S24 Elemental distribution of As-SNZVI in real groundwater. **Page S28**

Figure S25 As(III)-impacted hydrophobicity of SNZVI in real groundwater. **Page S29**

Figure S26 As(III)-impacted TCE reactivity of SNZVI in real groundwater. **Page S30**

Figure S27 As(III)-impacted surface area of SNZVI in real groundwater. **Page S31**

Table S1 Compositions of the used groundwater. **Page S32**

Table S2 Adsorption isotherm parameters of arsenite by SNZVI. **Page S33**

Table S3 Linear combination fitting results of XANES spectra at As K-edge. **Page S34**

References **Page S35**

Text S1 As Removal Kinetics and Capacity by SNZVI

The sulfur content of SNZVI did not affect the rate or extent of the As removal efficiency (close to 100%) at the low As/Fe ratio (i.e., 0.1 mg or 1 mg As per g SNZVI) (Figure S1a, c). The removal kinetics of arsenite by SNZVI with different S content followed a second-order-kinetic model (r^2>0.999) (Figure S1b, d, f). The similar removal efficiency and kinetics indicate the comparable performance of these materials for trace arsenite removal.

The adsorption isotherms of SNZVI materials for arsenite were performed to assess the impact of sulfur on the maximum capacity of arsenite. The equilibrium adsorption data could be well fitted with the Langmuir model for the materials without S or with low S (i.e. NZVI and 0.010 SNZVI) (Figure S1g). Detailed fitted parameters are shown in Table S1. In contrast, the adsorption of arsenite by the materials with high S content (i.e. 0.049 and 0.099 SNZVI) was better fitted with the Freundlich model (r^2=0.951 and 0.994, respectively) rather than the Langmuir model (r^2=0.539 and 0.842, respectively). While previous studies have proved that different metals (e.g., U, Au, and Ag) would have different distributions (e.g., cluster and encapsulation) over NZVI particles,[1, 2] the results here show that sulfidation of NZVI can alter the distribution and surface coverage of As(III), which would change the surface property and reactivity as discussed later.

In addition, the maximum adsorption capacity of arsenite by NZVI and SNZVI (0.010 $[S/Fe]_{particle}$) according to the Langmuir model could be up to 115 mg g^{-1} (i.e., 6.9 mg m^{-2}) and 135 mg g^{-1} (i.e., 12.1 mg m^{-2}), respectively. The higher capacity of 0.010 SNZVI than that of NZVI was probably because the 0.010 SNZVI material was the more reactive than NZVI,[3] generating more iron (hydr)oxides (i.e., FeOOH and ferrihydrite) on the surface with high affinity toward arsenite (Figure S8), and reducing

more arsenite to reduced forms (e.g., As^0). The reaction of S with arsenite to form As sulfides (e.g., realgar) would also improve the adsorption capacity of SNZVI as discussed later. The adsorption capacity of SNZVI was higher than most of functionalized iron/carbon materials either via direct adsorption[4] or pre-oxidation then adsorption process,[5, 6] indicating the good potential of SNZVI for the arsenite remediation.

Arsenite capacity follows the order of NZVI > 0.010 SNZVI > 0.099 SNZVI > 0.049 SNZVI at a relatively high As concentration, and this trend was well correlated with the hydrophobicity of these materials (Figure 3i), i.e., a hydrophobic SNZVI possessed a low removal capacity and rate for the hydrophilic arsenite ions. A similar trend was observed for the removals of Cr(VI) or Cd(II) by SNZVI materials, where the removal capacity of these metals was usually inversely proportional to the hydrophobicity of SNZVI.[3, 7-9] However, this trend was not consistent with a previous study where the removal efficiencies of As(III) by SNZVI materials (using an Fe^{2+} precursor and a $NaBH_4$/Fe molar ratio of 1.0) were all larger than that by NZVI.[10] This is because that different Fe precursors and $NaBH_4$/Fe would result in different physicochemical properties (e.g., Fe^0 content, S content and speciation, surface area, lattice constant, and electron transfer ability) of SNZVI materials,[11] which could possibly affect the performance of SNZVI materials. For example, our recent study found that the Brunauer-Emmett-Teller surface area of SNZVI synthesized by a low $NaBH_4$/Fe ratio (1.5) was ~5 times higher than that by a high $NaBH_4$/Fe ratio (5.0), while the surface area of NZVI was close at different $NaBH_4$/Fe ratios.[12] This is possibly one of the reasons for the discrepancy between this work and the previous study.[10]

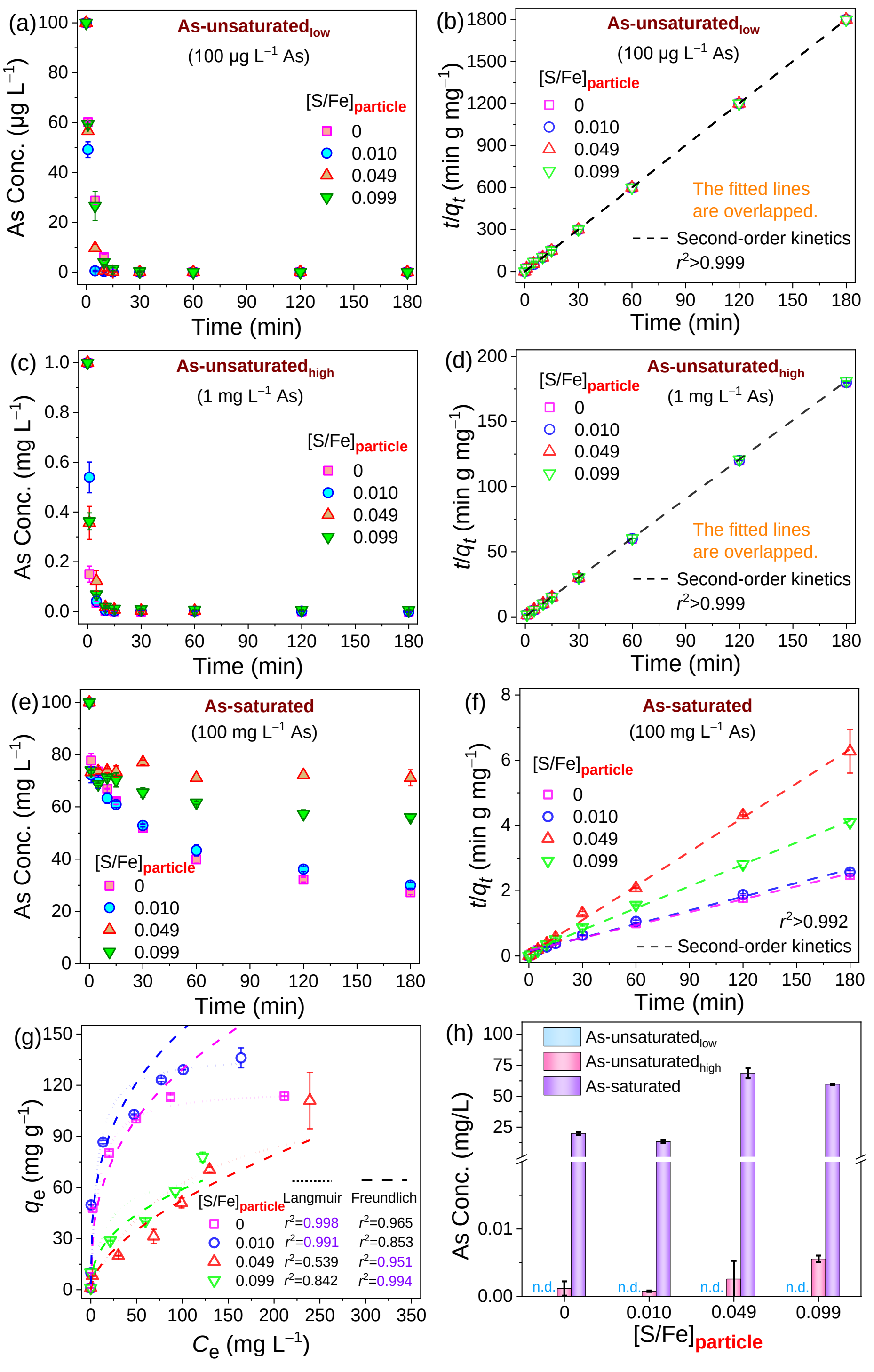

Figure S1 (a, c, and e) Arsenite removal performance and (b, d, and f) kinetics (during 180 min reaction) by SNZVI with different S contents. (g) Adsorption isotherm (after 24 h equilibrium) of As removal by SNZVI with different sulfur contents. (h) the equilibrium concentration of arsenite after 24-h reaction (totally 100 mg L^{-1} As(III)). Basic conditions: 1.0 g L^{-1} NZVI or SNZVI, initial pH = 5.5, T = 25±2 ^{o}C.

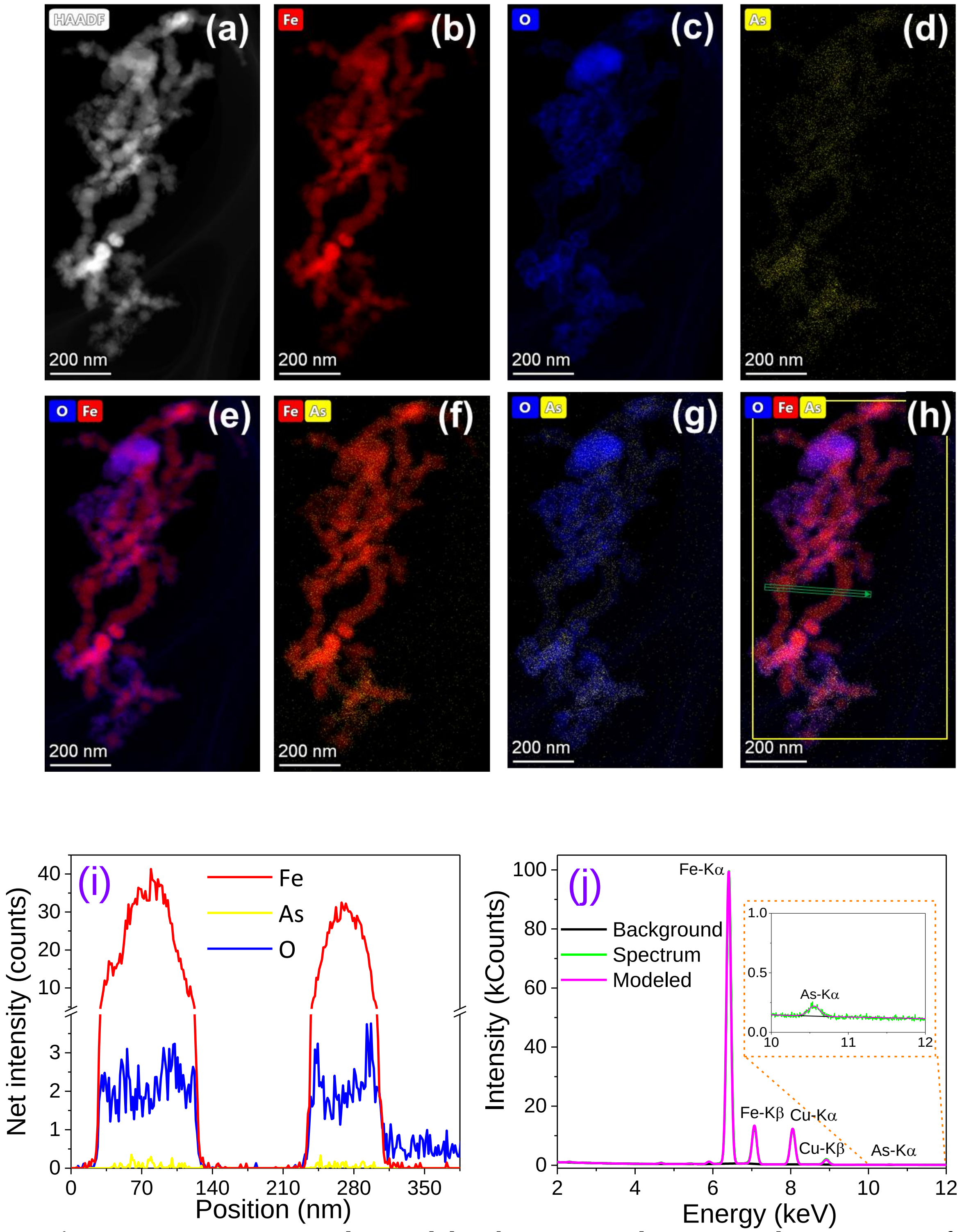

Figure S2 HAADF image, elemental distribution maps, line scan, and EDX spectra of As-reacted NZVI in the As-unsaturated$_{high}$ scenario (1.0 g L^{-1} NZVI, 1 mg L^{-1} As(III), initial pH = 5.5, T = 25±2 °C, reaction time = 24 h).

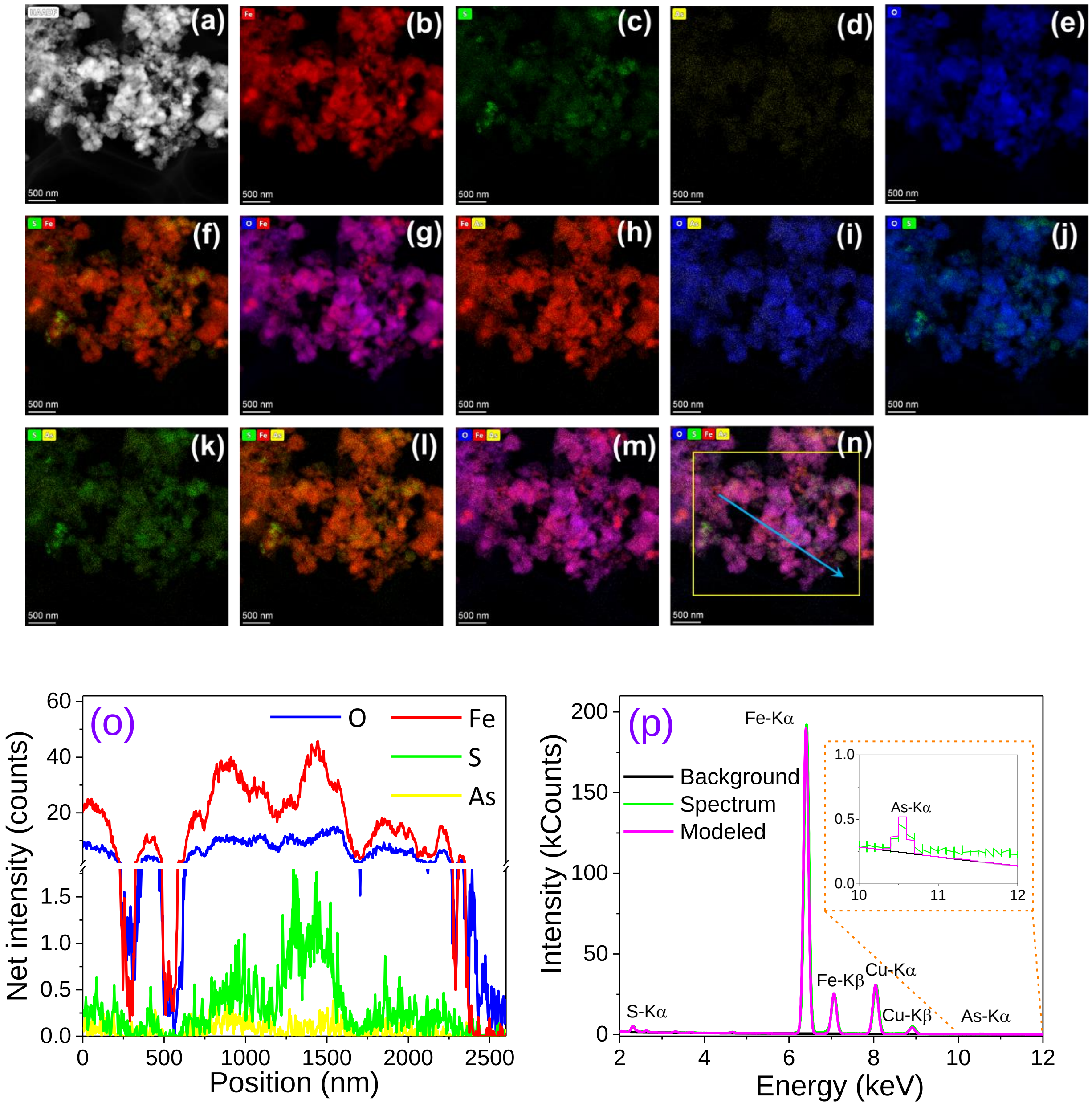

Figure S3 HAADF image, elemental distribution maps, line scan, and EDX spectra of As-reacted SNZVI ($[S/Fe]_{particle}$=0.010) in the As-unsaturated$_{high}$ scenario (1.0 g L^{-1} SNZVI, 1 mg L^{-1} As(III), initial pH = 5.5, T = 25±2 °C, reaction time = 24 h).

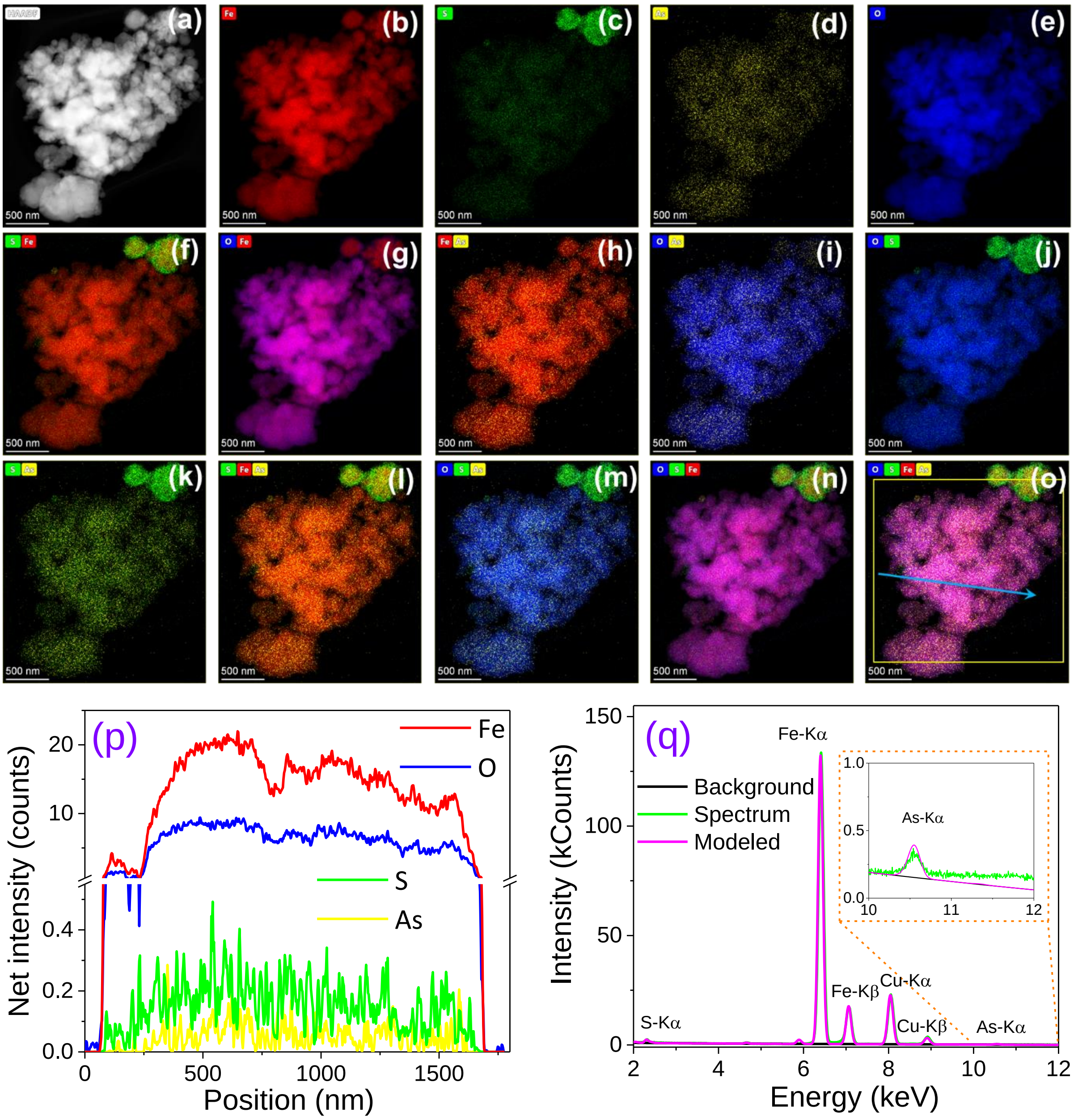

Figure S4 HADAF image, elemental distribution maps, line scan, and EDX spectra of As-reacted SNZVI ($[S/Fe]_{particle}$=0.049) in the As-unsaturated$_{high}$ scenario (1.0 g L^{-1} SNZVI, 1 mg L^{-1} As(III), initial pH = 5.5, T = 25±2 °C, reaction time = 24 h).

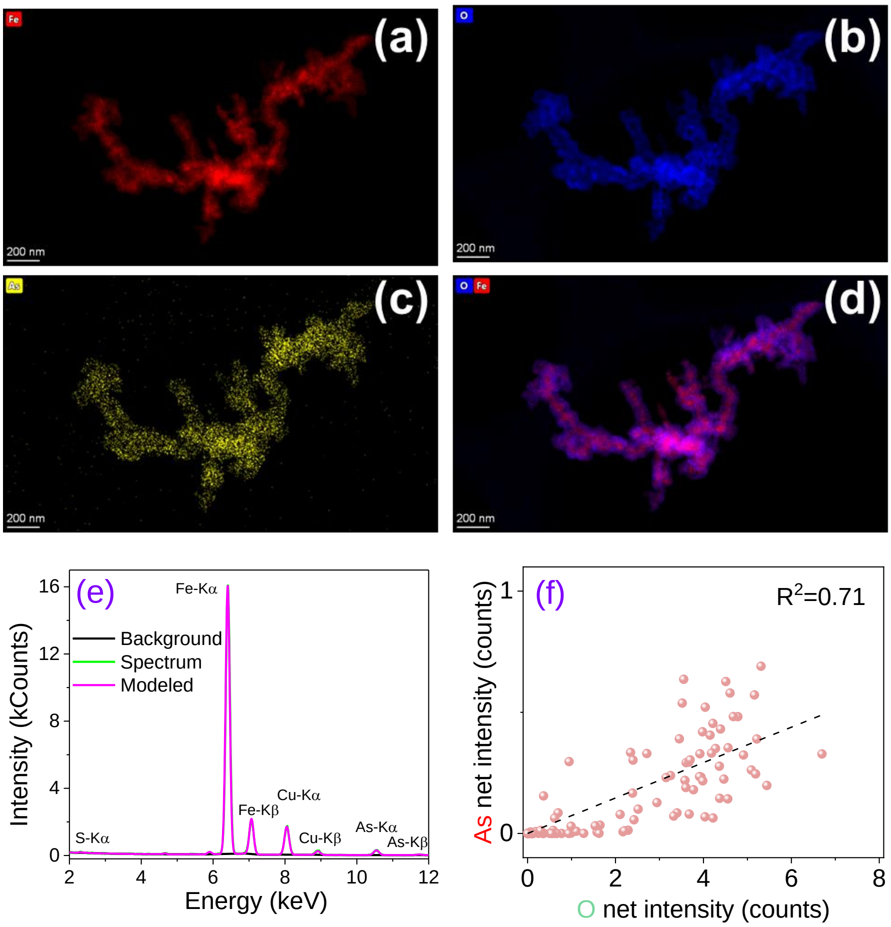

Figure S5 Elemental distribution maps and EDX spectra of As-reacted NZVI in the As-saturated scenario (1.0 g L^{-1} NZVI, 100 mg L^{-1} As(III), initial pH = 5.5, T = 25±2 ^{o}C, reaction time = 24 h).

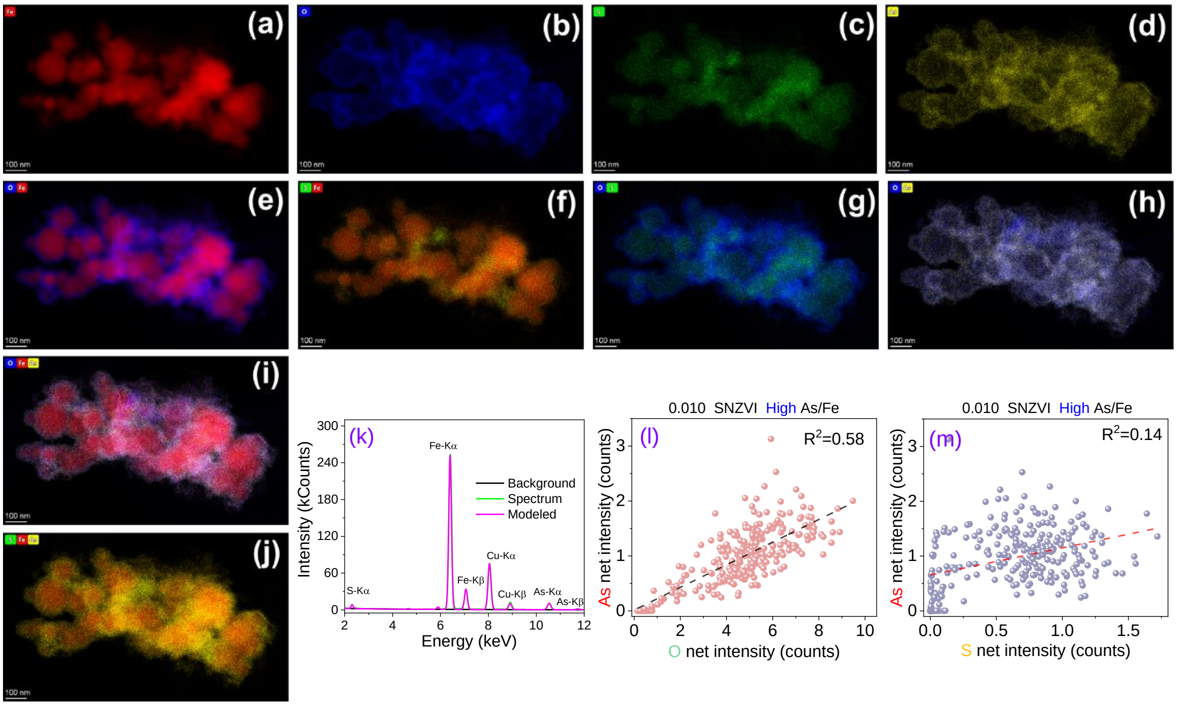

Figure S6 Elemental distribution maps, EDX spectra and intensity correlation of As-reacted SNZVI ($[S/Fe]_{particle}$=0.010) in the As-saturated scenario (1.0 g L^{-1} SNZVI, 100 mg L^{-1} As(III), initial pH = 5.5, T = 25±2 ^{o}C, reaction time = 24 h).

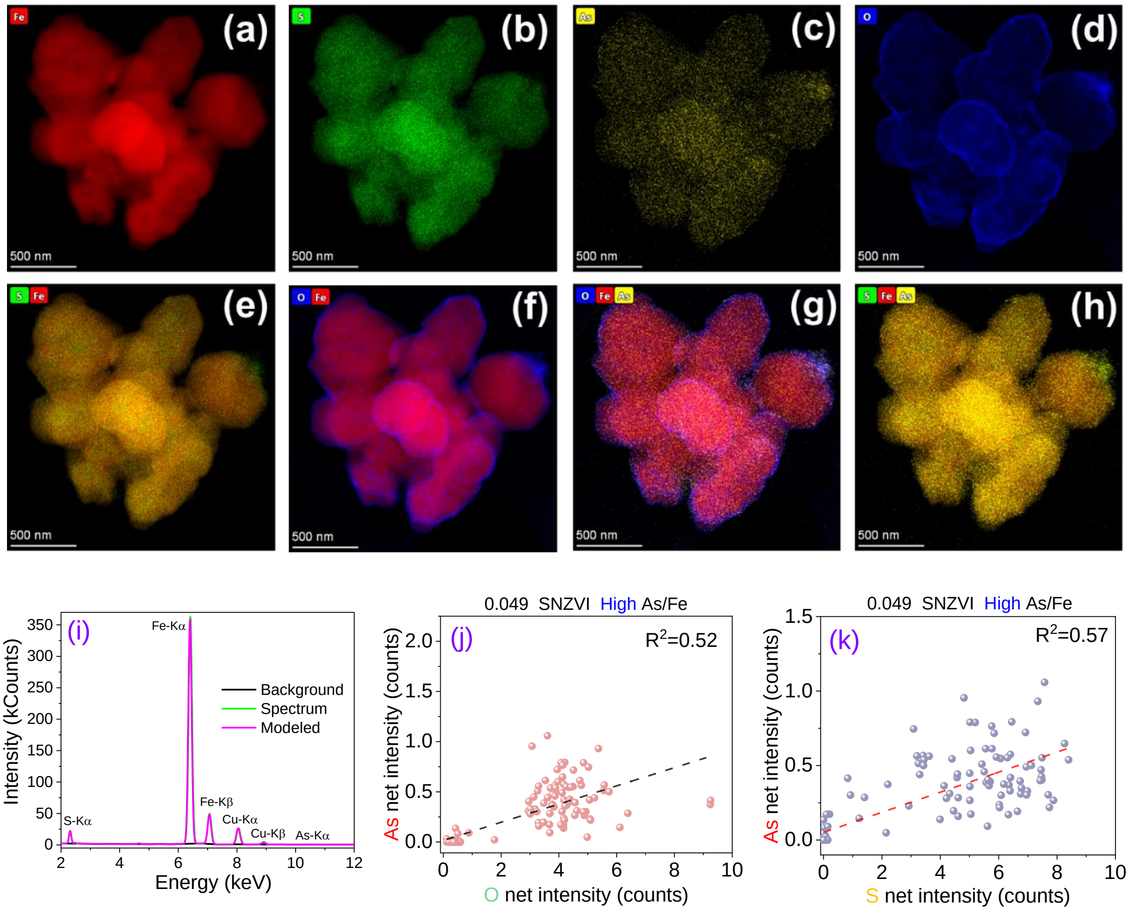

Figure S7 Elemental distribution maps, EDX spectra and intensity correlation of As-reacted SNZVI ($[S/Fe]_{particle}$=0.049) in the As-saturated scenario (1.0 g L^{-1} SNZVI, 100 mg L^{-1} As(III), initial pH = 5.5, T = 25±2 °C, reaction time = 24 h).

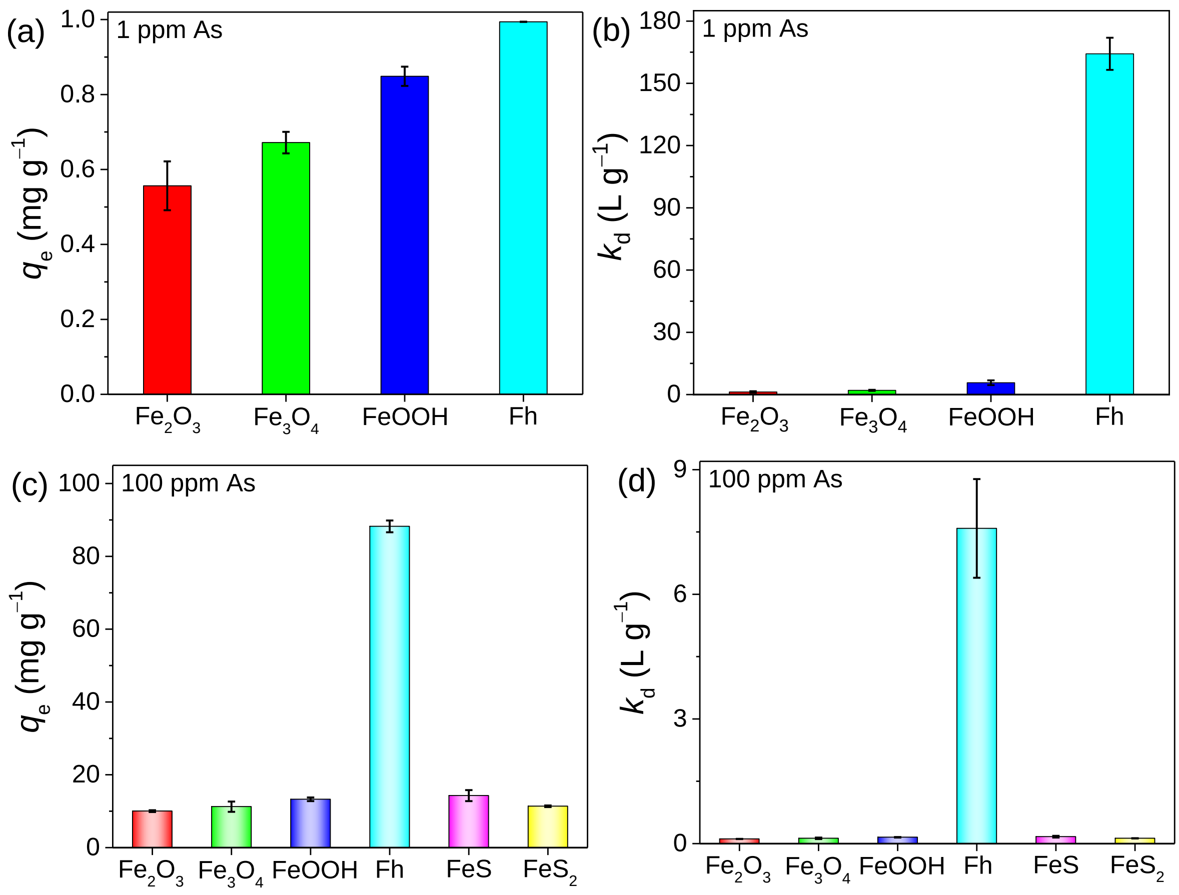

Figure S8 Adsorption capacity (q_e) and solid water distribution coefficients (k_d) of As by different iron and sulfur compounds (1.0 g L^{-1} material, 1 or totally 100 mg L^{-1} As(III), initial pH = 5.5, T = 25±2 ^oC).

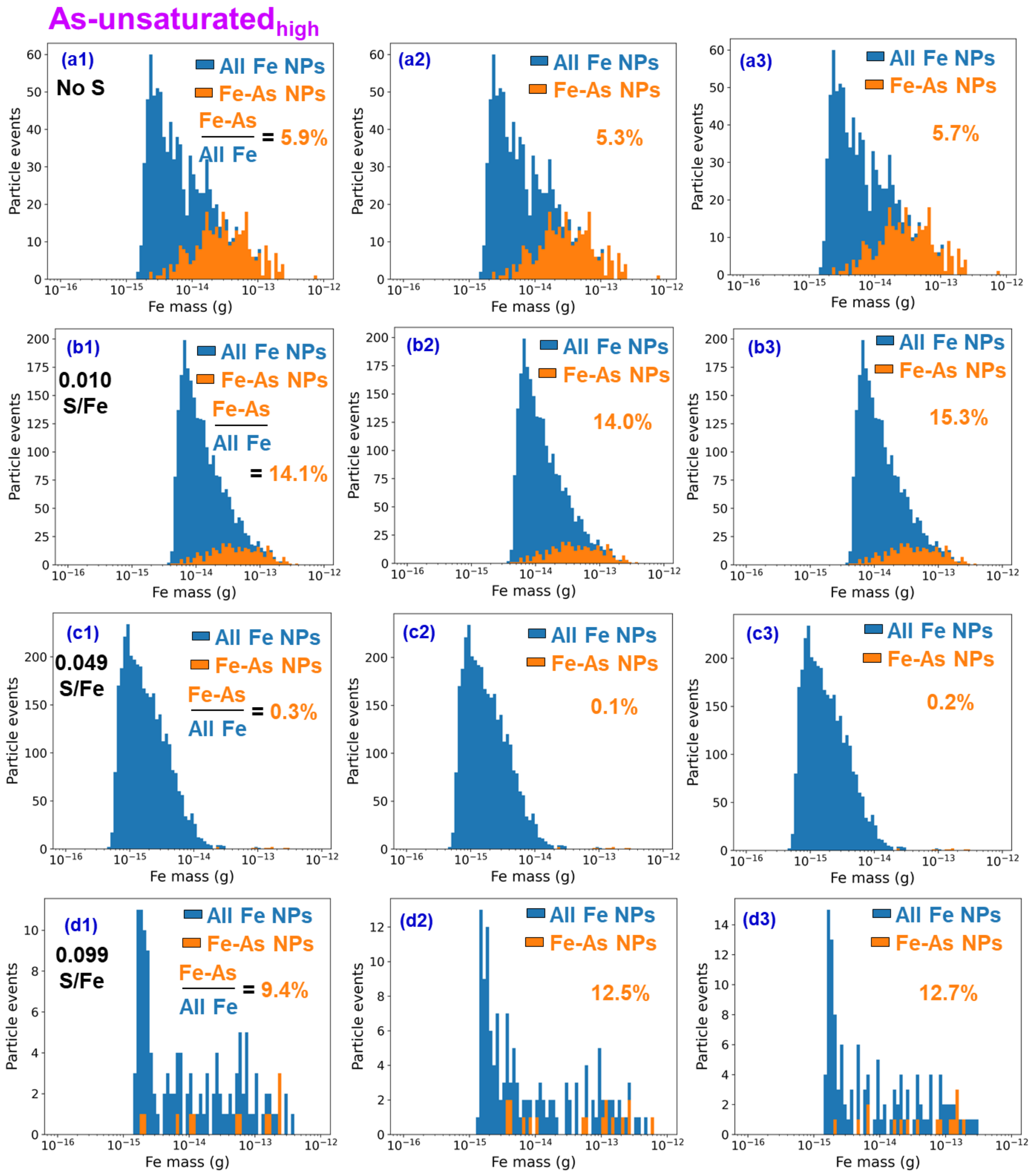

Figure S9 spICP-TOF-MS analysis (triplicates) of Fe mass distribution and the percentage of As-bearing Fe NPs over the total Fe NPs in the As-unsaturated$_{high}$ scenario. (a) As-reacted NZVI, (b) As-reacted 0.010 SNZVI, (c) As-reacted 0.049 SNZVI, and (d) As-reacted 0.099 SNZVI (1.0 g L^{-1} NZVI or SNZVI, 1 mg L^{-1} As(III), initial pH = 5.5, T = 25±2 ºC).

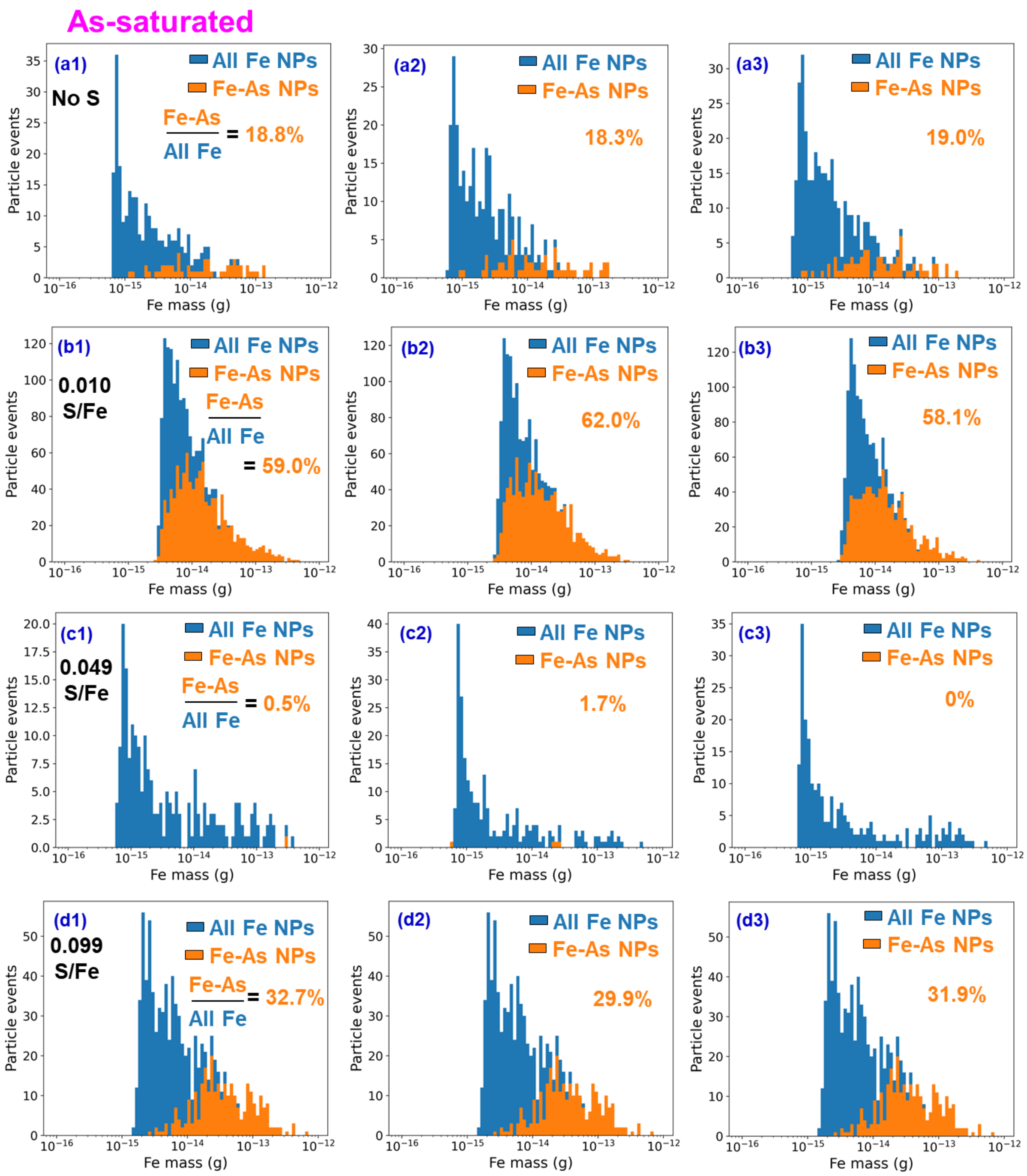

Figure S10 spICP-TOF-MS analysis (triplicates) of Fe mass distribution and the percentage of As-bearing Fe NPs over the total Fe NPs in the As-saturated scenario. (a) As-reacted NZVI, (b) As-reacted 0.010 SNZVI, (c) As-reacted 0.049 SNZVI, and (d) As-reacted 0.099 SNZVI (1.0 g L^{-1} NZVI or SNZVI, totally 100 mg L^{-1} As(III), initial pH = 5.5, T = 25±2 ^{o}C).

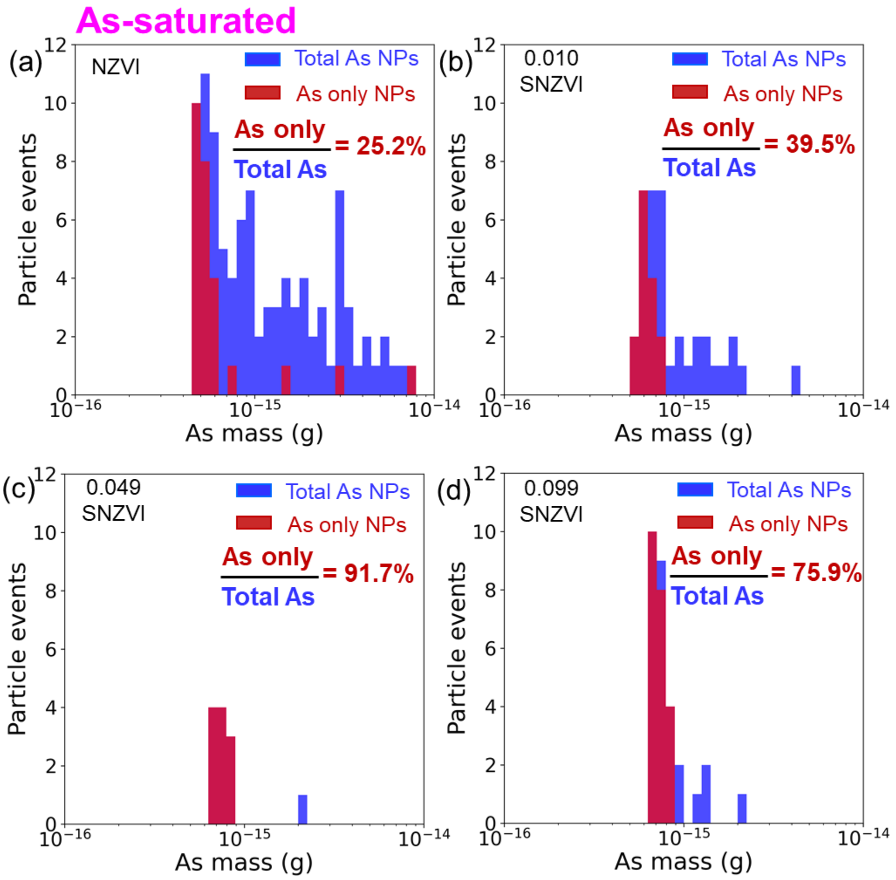

Figure S11 spICP-TOF-MS analysis of As mass distribution and the percentage of "As only" NPs in the As-saturated scenario. (a) As-reacted NZVI, (b) As-reacted 0.010 SNZVI, (c) As-reacted 0.049 SNZVI, and (d) As-reacted 0.099 SNZVI (1.0 g L^{-1} NZVI or SNZVI, totally 100 mg L^{-1} As(III), initial pH = 5.5, T = 25±2 ^{o}C).

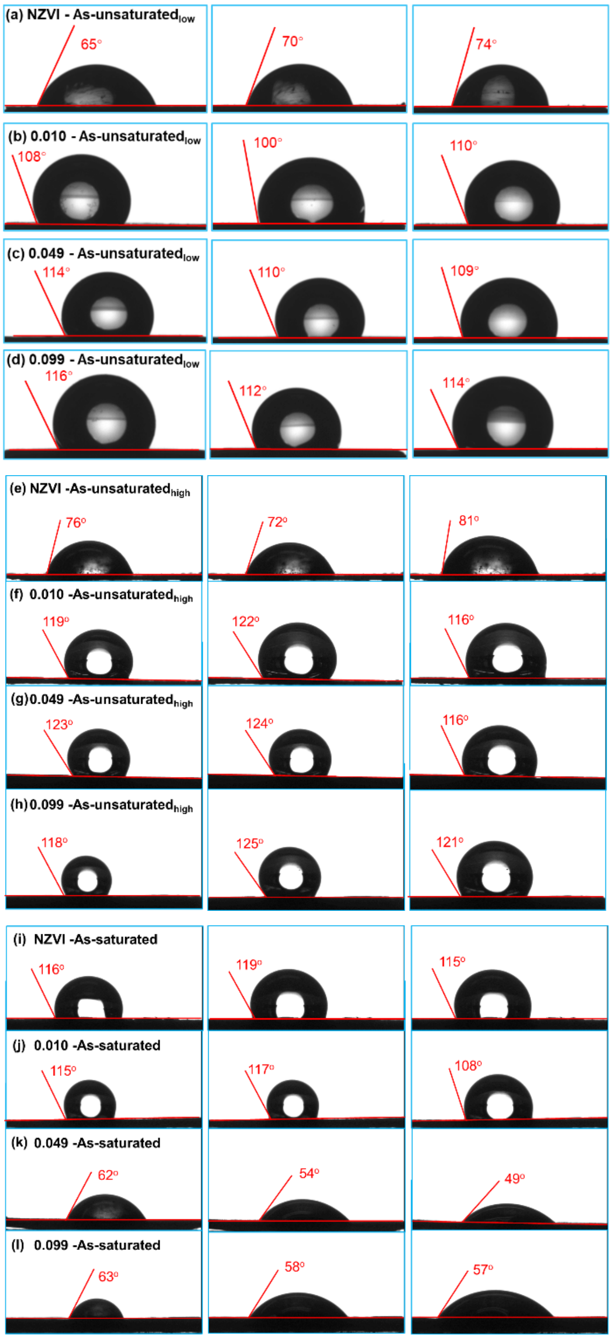

Figure S12 Images of water contact angle measurements (triplicates) for As-reacted SNZVI pellets in air (1.0 g L^{-1} NZVI or SNZVI, initial pH = 5.5, T = 25±2 °C, reaction time = 24 h. An addition of 100 µg L^{-1}, 1 mg L^{-1} and totally 100 mg L^{-1} As(III) represents the As-unsaturated$_{low}$, As-unsaturated$_{high}$, and As-saturated scenario, respectively).

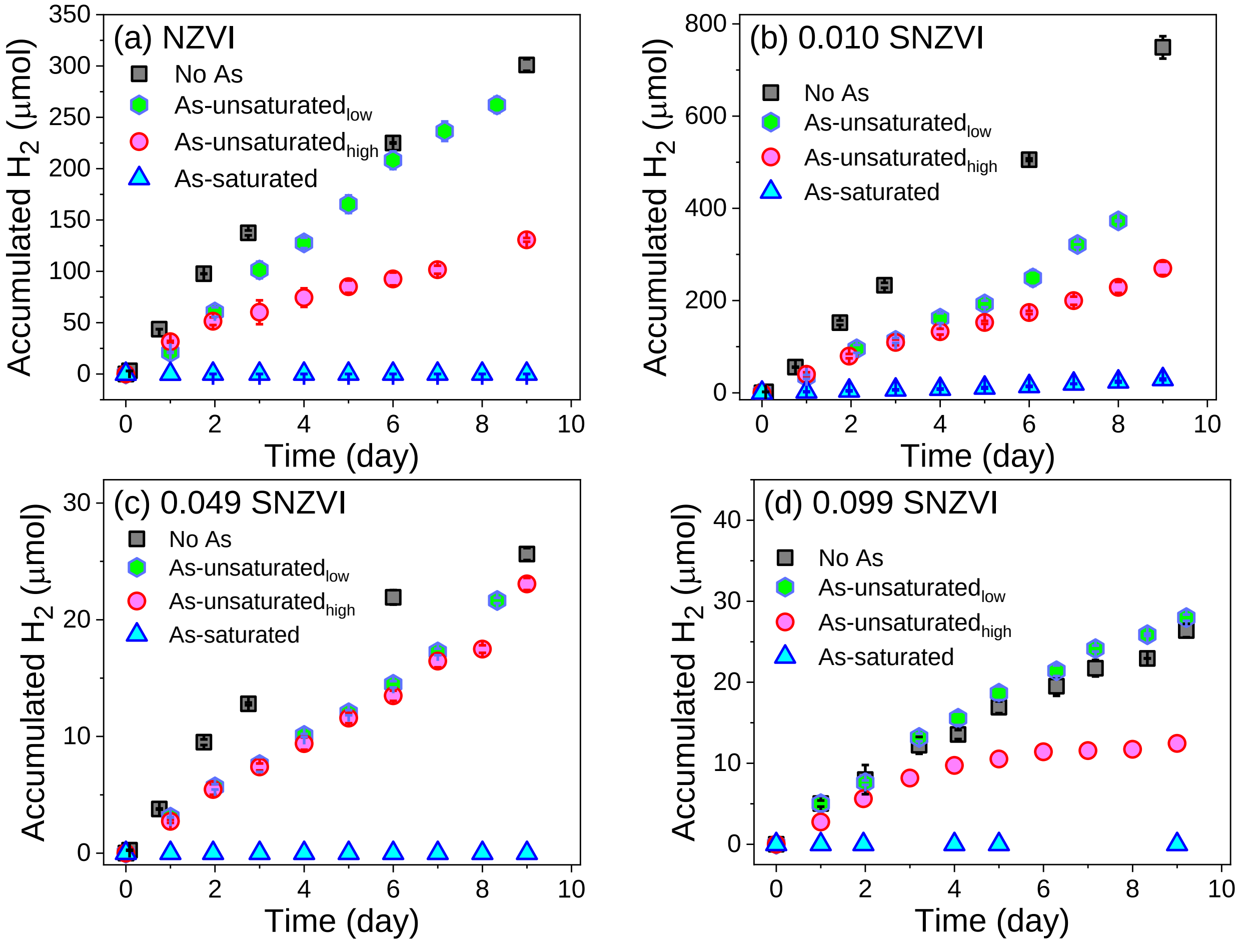

Figure S13 Impact of co-existed As(III) on the reactivity of NZVI and SNZVI with water (1.0 g L^{-1} NZVI or SNZVI, 100 μg L^{-1} or 1 mg L^{-1} or 100 mg L^{-1} As(III), initial pH = 5.5, T = 25±2 ^{o}C).

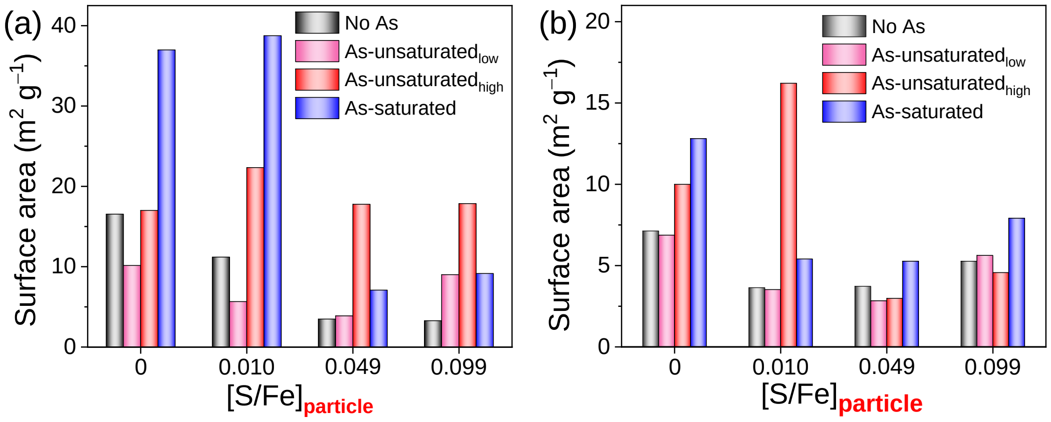

Figure S14 Brunauer-Emmett-Teller surface area of fresh and As-reacted SNZVI materials for (a) water and TCE reactivity, (b) FF reactivity.

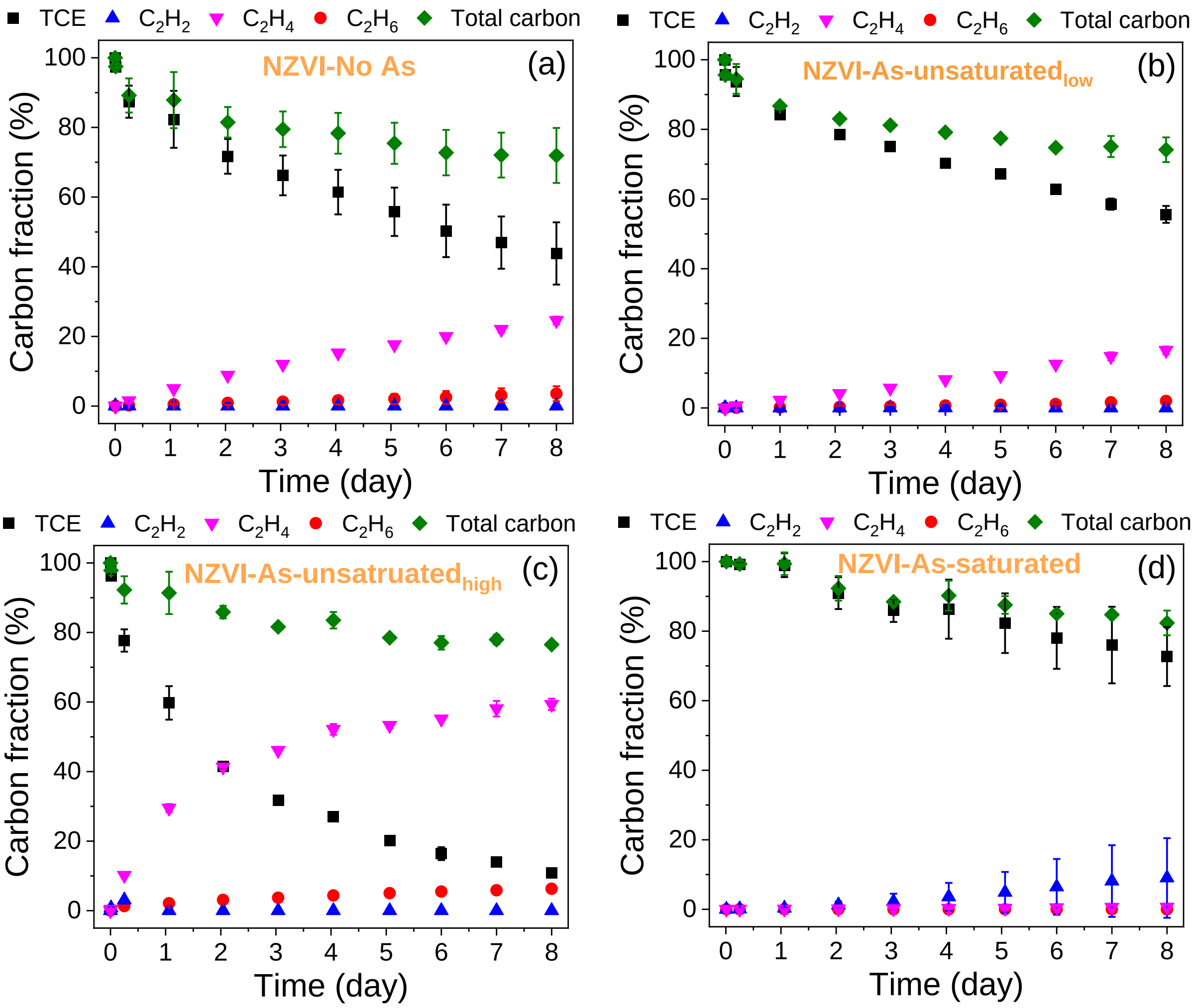

Figure S15 Impact of co-existed arsenite on the TCE removal by NZVI in the (a) no As, (b) As-unsaturated$_{low}$, (c) As-unsatrated$_{high}$, and (d) As-saturated scenarios (1.0 g L^{-1} NZVI, 70 μM TCE, 100 μg L^{-1} or 1 mg L^{-1} or totally 100 mg L^{-1} As(III), initial pH = 5.5, T = 25±2 ^{o}C).

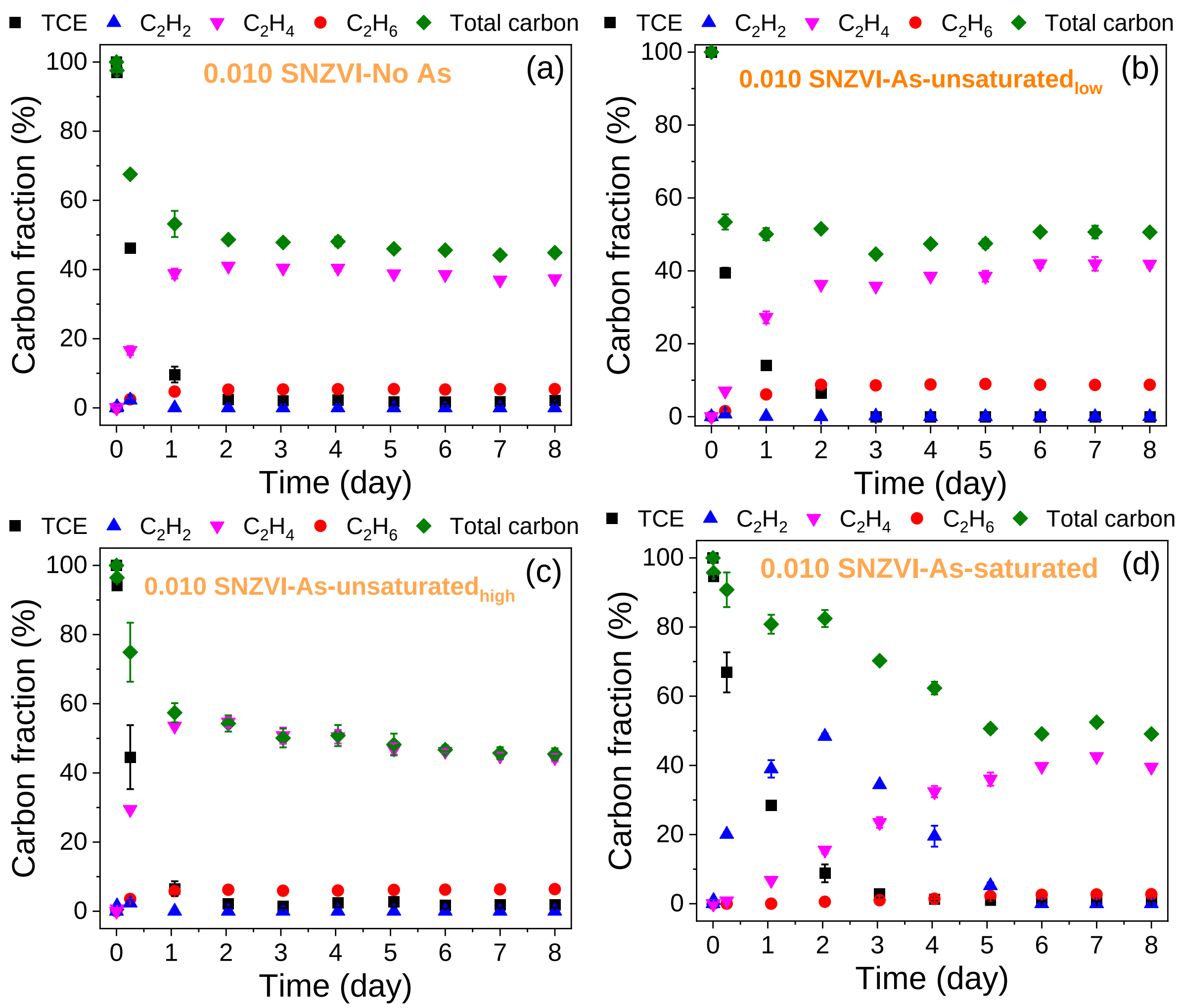

Figure S16 Impact of co-existed arsenite on the TCE removal by 0.010 SNZVI in the (a) no As, (b) As-unsaturated$_{\mathrm{low}}$, (c) As-unsatrated$_{\mathrm{high}}$, and (d) As-saturated scenarios (1.0 g L^{-1} SNZVI, 70 μM TCE, 100 μg L^{-1} or 1 mg L^{-1} or totally 100 mg L^{-1} As(III), initial pH = 5.5, T = 25±2 ºC)

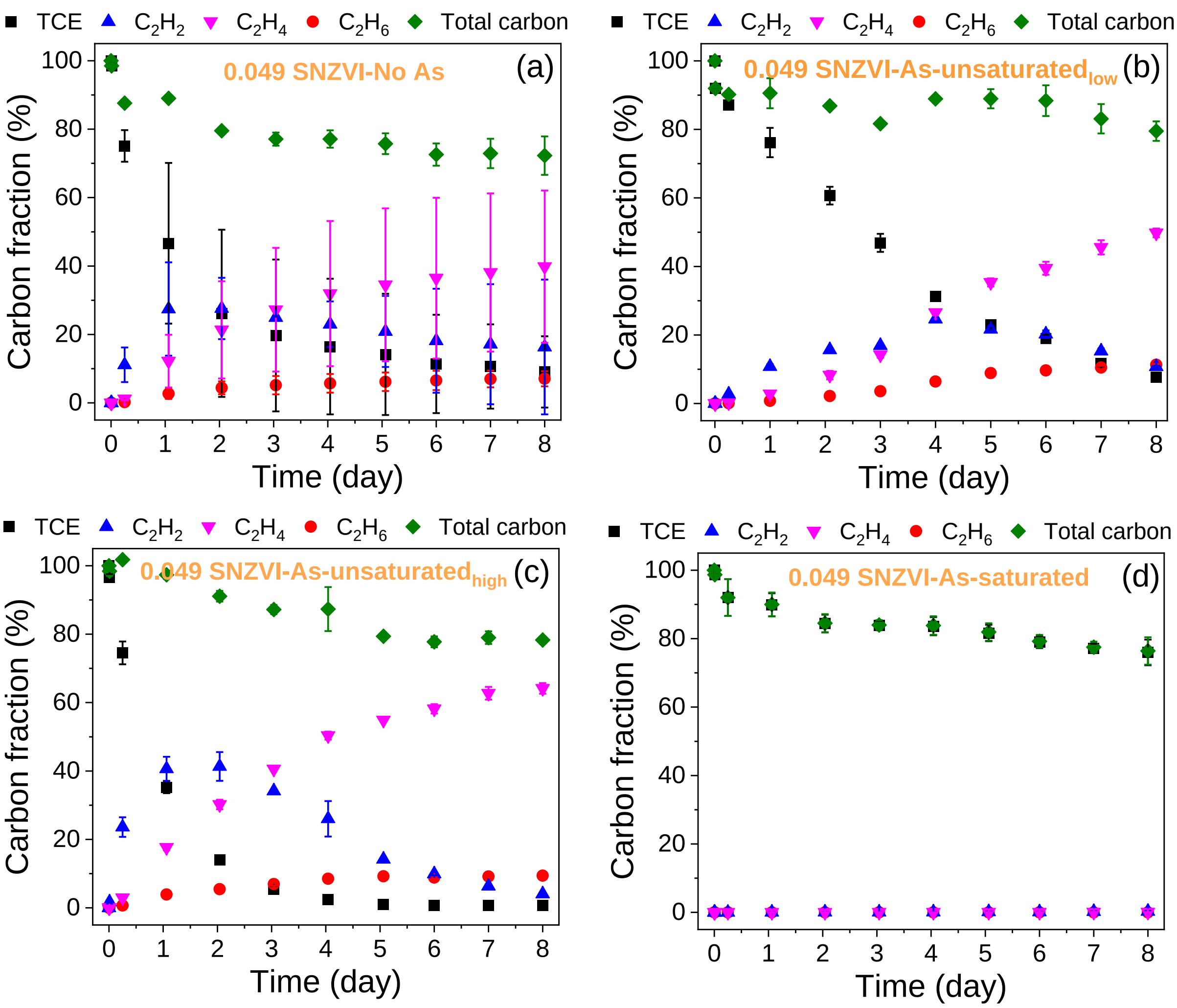

Figure S17 Impact of co-existed arsenite on the TCE removal by 0.049 SNZVI in the (a) no As, (b) As-unsaturated$_{low}$, (c) As-unsatrated$_{high}$, and (d) As-saturated scenarios (1.0 g L^{-1} SNZVI, 70 μM TCE, 100 μg L^{-1} or 1 mg L^{-1} or totally 100 mg L^{-1} As(III), initial pH = 5.5, T = 25±2 °C)

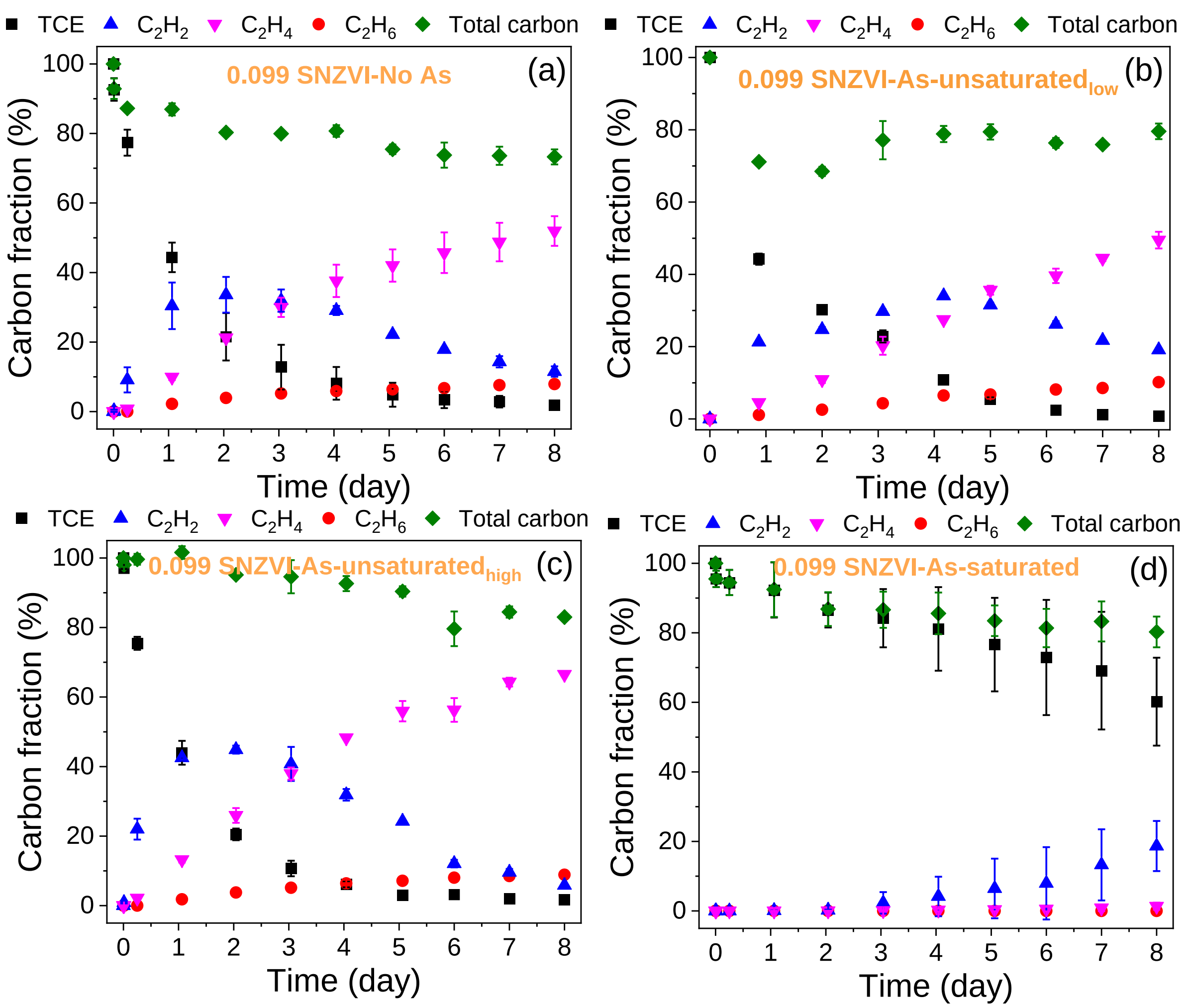

Figure S18 Impact of co-existed arsenite on the TCE removal by 0.099 SNZVI in the (a) no As, (b) As-unsaturated$_{low}$, (c) As-unsatrated$_{high}$, and (d) As-saturated scenarios (1.0 g L^{-1} SNZVI, 70 μM TCE, 100 μg L^{-1} or 1 mg L^{-1} or totally 100 mg L^{-1} As(III), initial pH = 5.5, T = 25±2 ^{o}C)

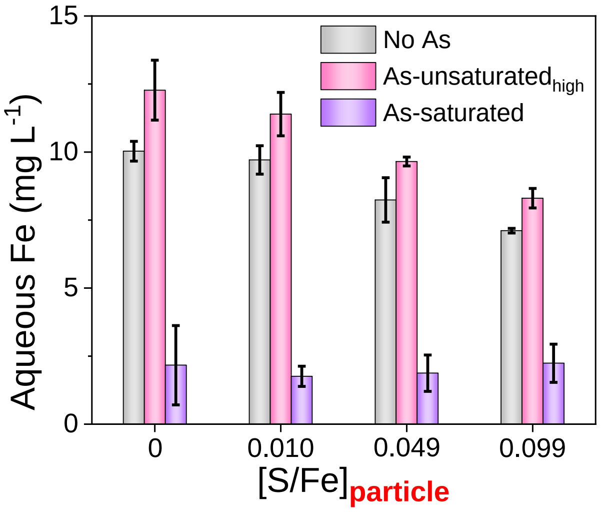

Figure S19 The concentration of aqueous Fe after 8-day TCE reaction by SNZVI (1.0 g L^{-1} SNZVI, 70 μM TCE, 1 mg L^{-1} or totally 100 mg L^{-1} As(III), initial pH = 5.5, T = 25±2 ^{o}C)

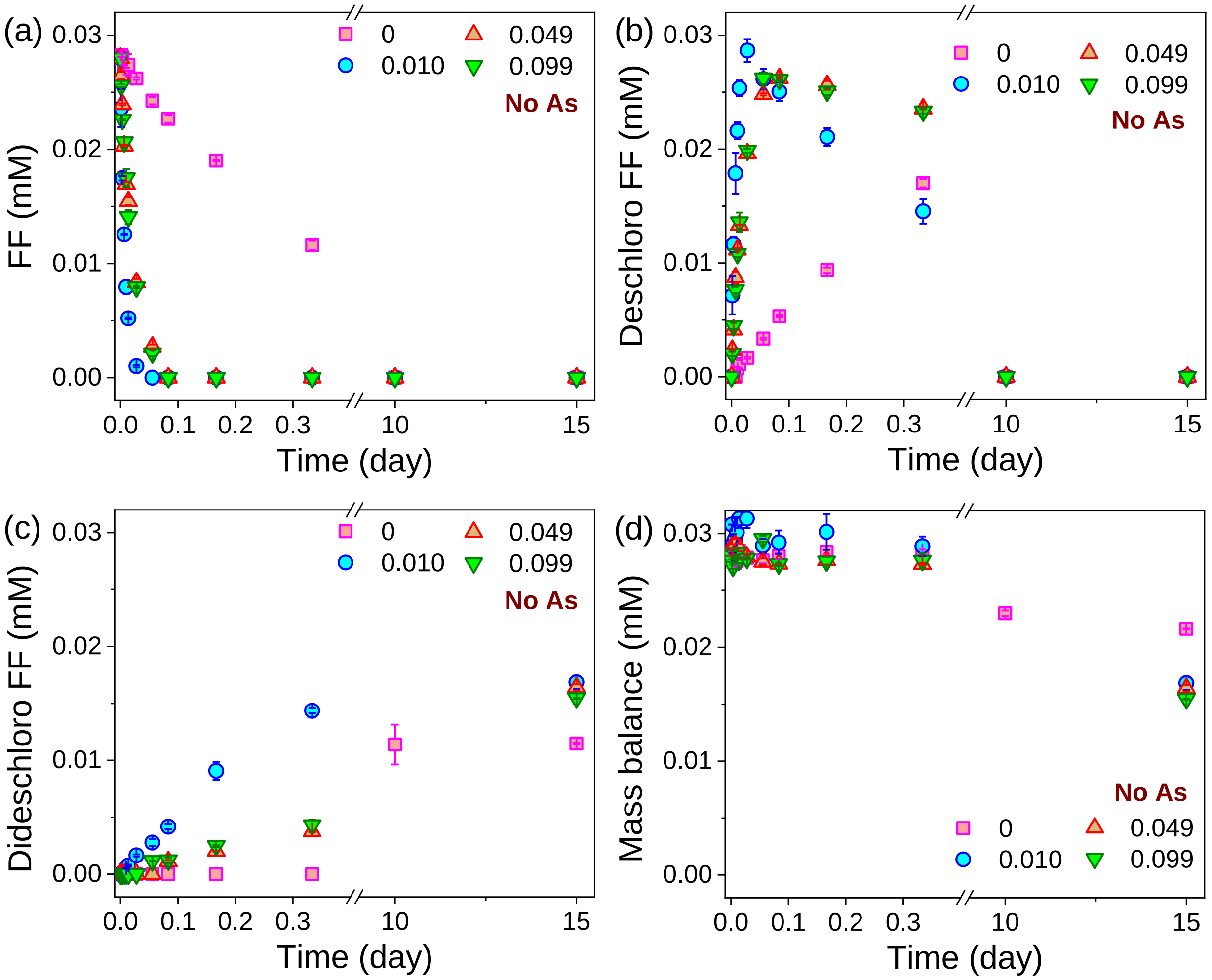

Figure S20 Impact of co-existed arsenite on the FF removal by NZVI or SNZVI with different S content in the no As scenarios (1.0 g L^{-1} NZVI or SNZVI, 0.028 mM FF, initial pH = 5.5, T = 25±2 °C)

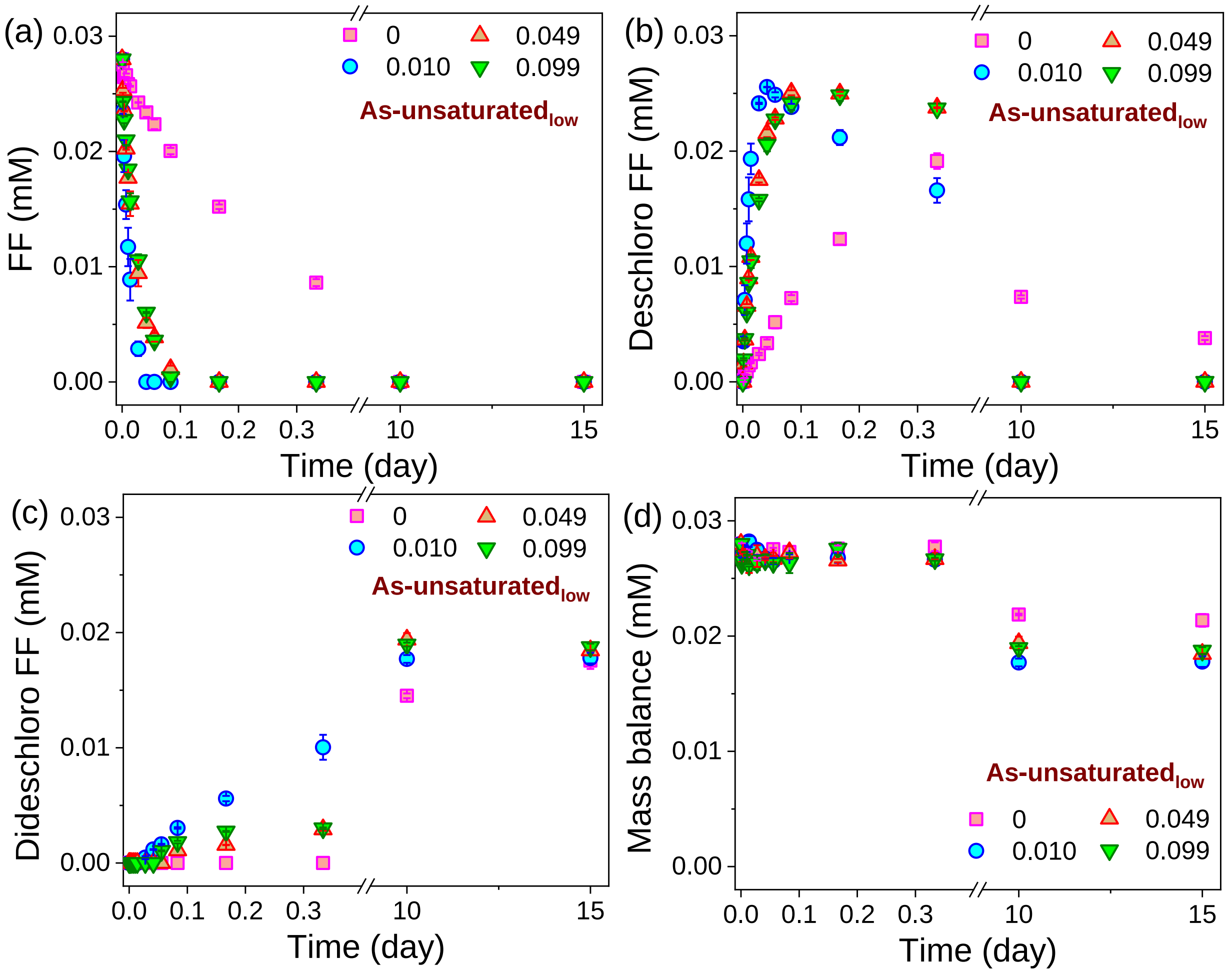

Figure S21 Impact of co-existed arsenite on the FF removal by NZVI or SNZVI with different S content in the As-unsaturated$_{low}$ scenarios (1.0 g L^{-1} NZVI or SNZVI, 0.028 mM FF, 100 μg L^{-1} As(III), initial pH = 5.5, T = 25±2 °C)

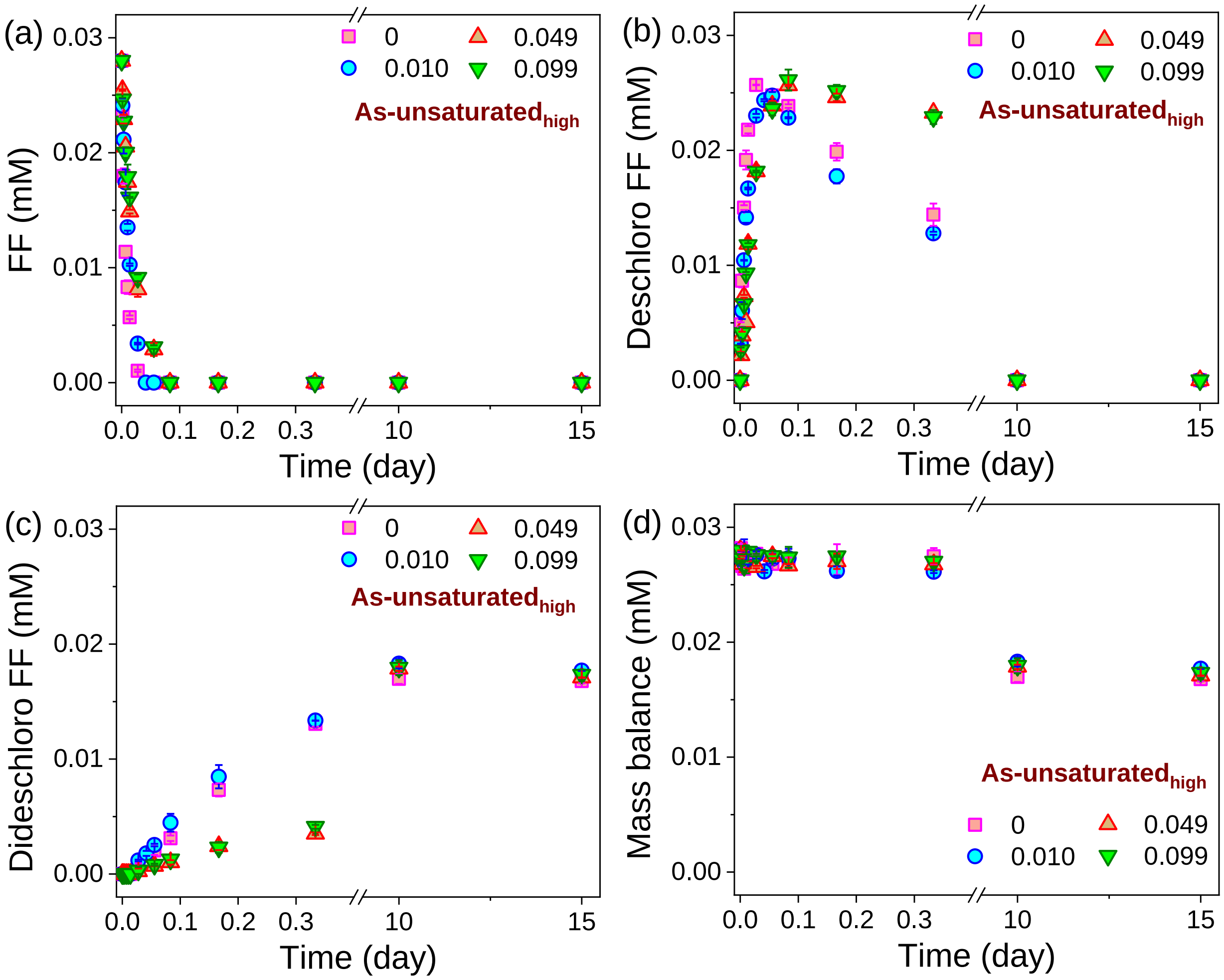

Figure S22 Impact of co-existed arsenite on the FF removal by NZVI or SNZVI with different S content in the As-unsaturated$_{high}$ scenarios (1.0 g L^{-1} NZVI or SNZVI, 0.028 mM FF, 1 mg L^{-1} As(III), initial pH = 5.5, T = 25±2 ^{o}C)

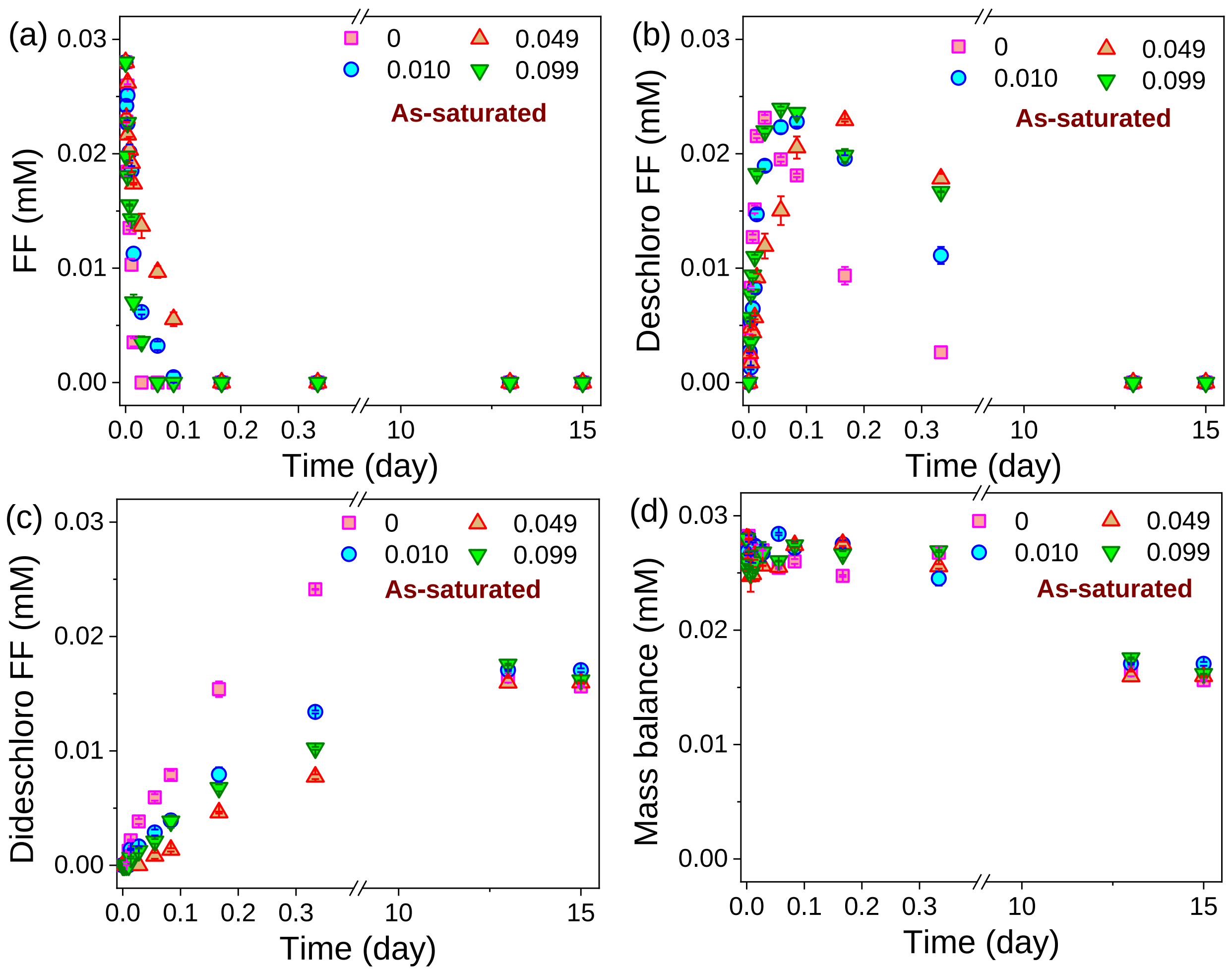

Figure S23 Impact of co-existed arsenite on the FF removal by NZVI or SNZVI with different S content in the As-saturated scenarios (1.0 g L^{-1} NZVI or SNZVI, 0.028 mM FF, totally 100 mg L^{-1} As(III), initial pH = 5.5, T = 25±2 °C).

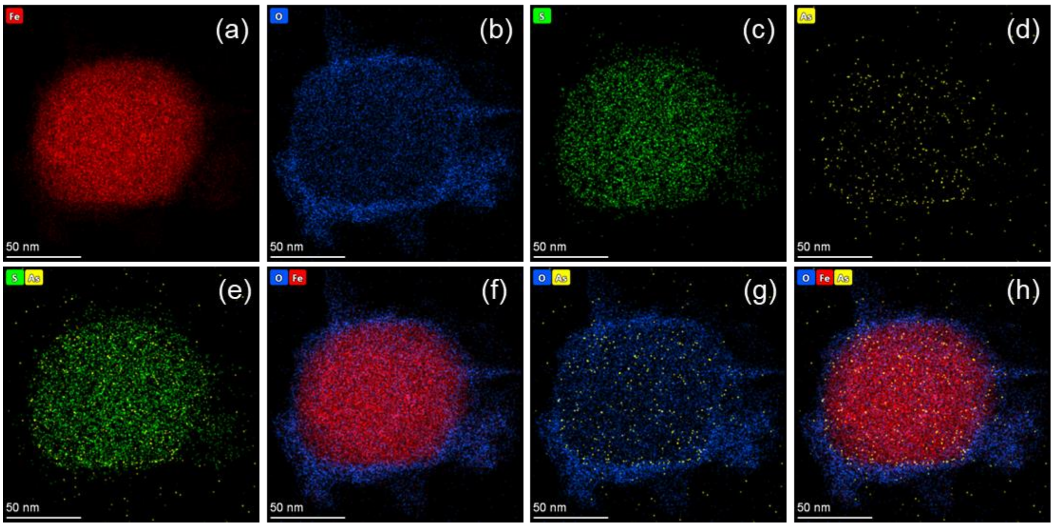

Figure S24 Elemental distribution maps of As-reacted SNZVI ($[S/Fe]_{particle}$=0.049) in the real groundwater (1.0 g L^{-1} SNZVI, 1 mg L^{-1} As(III), initial pH = 5.5, T = 25±2 ^{o}C, reaction time = 24 h).

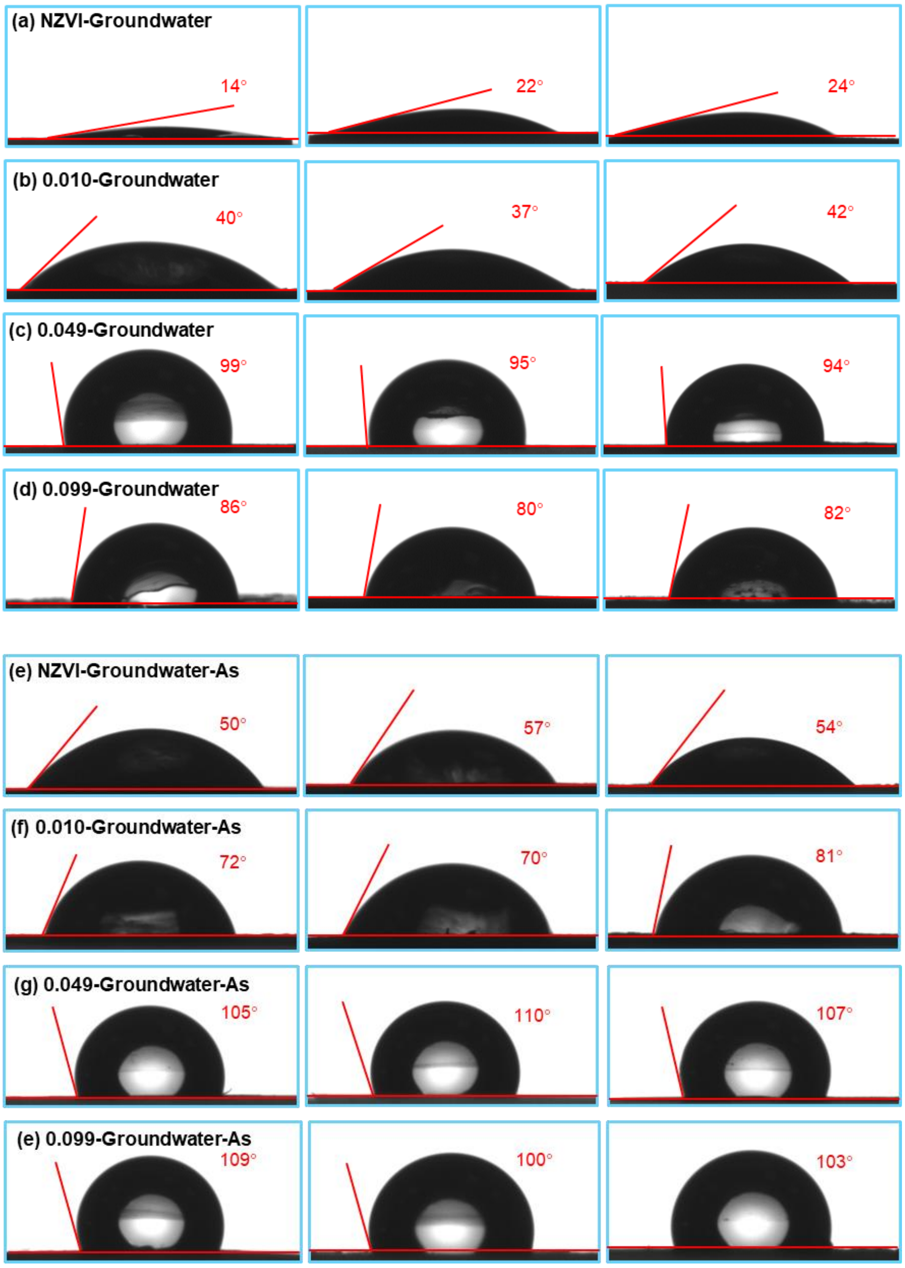

Figure S25 Impact of 100 μg L^{-1} As(III) in the real groundwater on the water contact angle (triplicates) measurements for As-reacted SNZVI pellets in air (100 μg L^{-1} As(III),1.0 g L^{-1} NZVI or SNZVI, initial pH = 5.5, T = 25±2 °C, reaction time = 24 h).

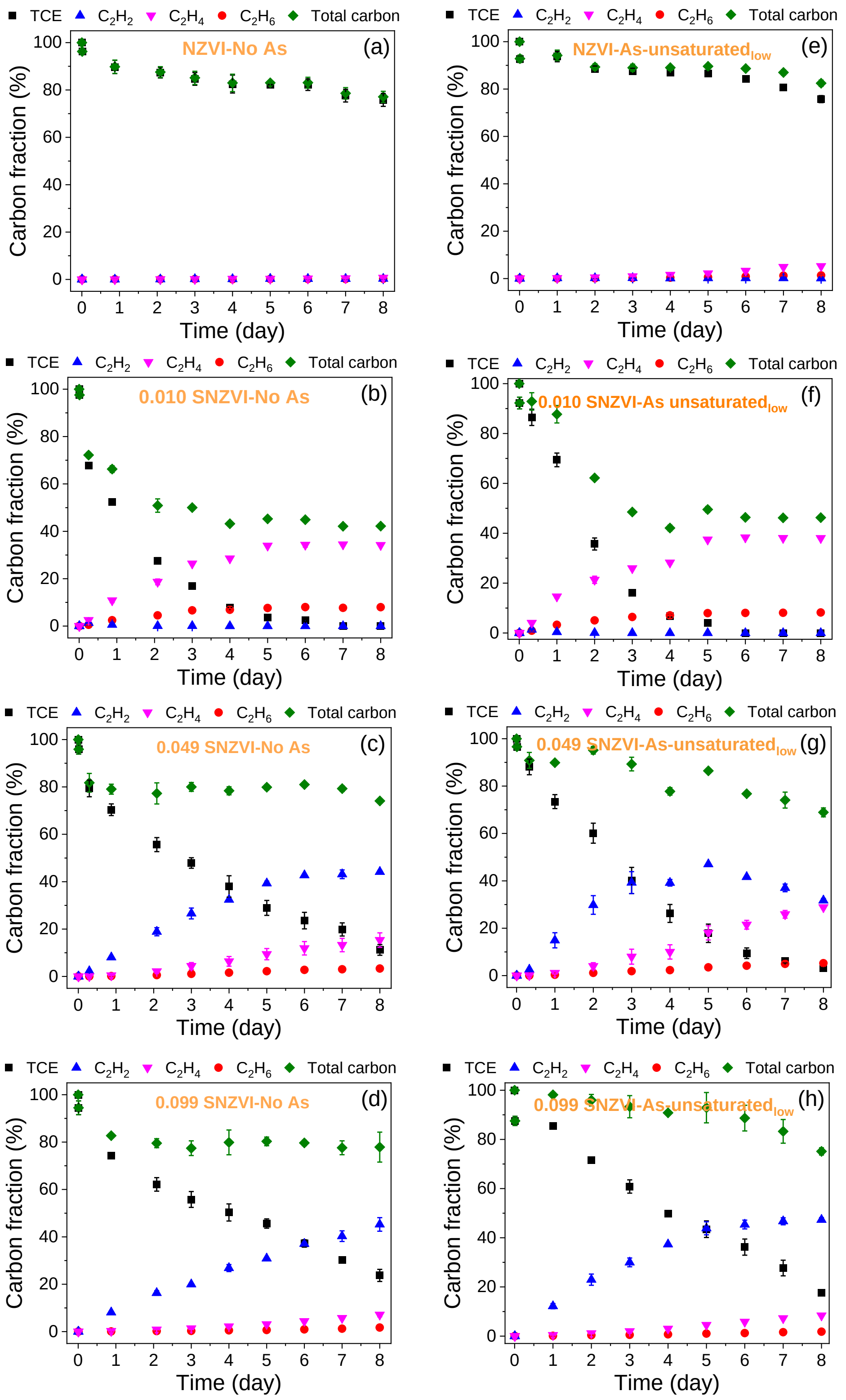

Figure S26 Impact of co-existed 100 μg L^{-1} As(III) on the TCE removal by SNZVI in the real groundwater (1.0 g L^{-1} SNZVI, 70 μM TCE, initial pH = 5.5, T = 25±2 °C).

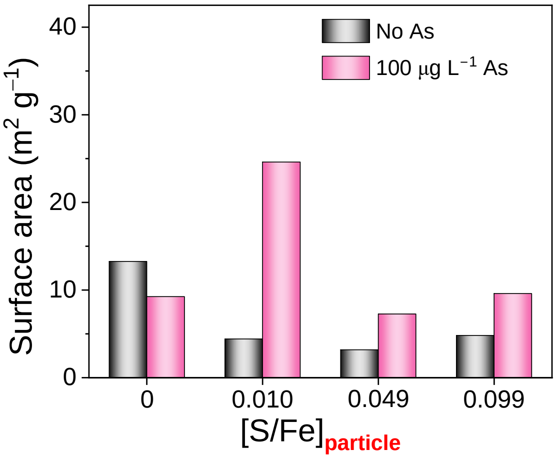

Figure S27 Brunauer-Emmett-Teller surface area of fresh and As-reacted SNZVI materials for assess the impacts of 100 μg L^{-1} As(III) on TCE reactivity in the real groundwater (1.0 g L^{-1} SNZVI, 70 μM TCE, initial pH = 5.5, T = 25±2 ^{o}C).

Table S1 Compositions of the groundwater used in this study.[13]

Component	Value
pH	7.2
Oxidation-reduction potential (mV)	143
Ionic strength (mol L^{-1})	32.2
TOC (mg L^{-1})	0.2
TC (mg L^{-1})	46.6
Na^{+} (mg L^{-1})	33.2
Ca^{2+} (mg L^{-1})	3.7
Mg^{2+} (mg L^{-1})	3.0
NO_3^{-} (mg L^{-1})	8.2
Cl^{-} (mg L^{-1})	22.3
SO_4^{2-} (mg L^{-1})	26.1

Table S2 Adsorption isotherm parameters of As(III) by SNZVI (1.0 g L^{-1} NZVI or SNZVI, initial pH = 5.5, T = 25±2 °C).

Materials	Langmuir model			Freundlich model		
	q_m (mg g^{-1})	K_L (L g^{-1})	r^2	n	K_F (mg g^{-1} (mg $L^{-1})^n$)	r^2
NZVI	114.9	0.293	0.998	2.72	23.8	0.965
0.010 SNZVI	135.1	0.233	0.991	3.07	33.9	0.853
0.049 SNZVI	125.0	0.010	0.539	2.68	8.7	0.951
0.099 SNZVI	63.7	0.042	0.920	3.00	9.8	0.818

Table S3 Linear combination fitting results of XANES spectra in R space at As K-edge.

Samples		As(III) (%)	As(V)-Fh (%)	As(II)-S (%)	As^0 (%)	R-factor	Chi-square	Reduced Chi-square
NZVI	Low As/Fe	44.6	55.4	0	0	0.002	0.068	0.001
0.010 SNZVI		39.3	36.0	24.7	0	0.006	0.168	0.002
0.049 SNZVI		39.7	27.3	33.0	0	0.005	0.027	0.027
NZVI	High As/Fe	51.0	21.1	0	27.9	0.002	0.070	0.001
0.010 SNZVI		51.9	17.8	0	30.3	0.002	0.054	0.001
0.049 SNZVI		62.8	18.5	0	18.7	0.003	0.104	0.001

References:

(1) Ling, L.; Zhang, W. Enrichment and Encapsulation of Uranium with Iron Nanoparticle. *J. Am. Chem. Soc.* **2015**, *137*, 2788-2791.

(2) Li, J.; Guan, X.; Zhang, W. Architectural Genesis of Metal(loid)S with Iron Nanoparticle in Water. *Environ. Sci. Technol.* **2021**, *55*, 12801-12808.

(3) Xu, J.; Avellan, A.; Li, H.; Liu, X.; Noël, V.; Lou, Z.; Wang, Y.; Kaegi, R.; Henkelman, G.; Lowry, G.V. Sulfur Loading and Speciation Control the Hydrophobicity, Electron Transfer, Reactivity, and Selectivity of Sulfidized Nanoscale Zerovalent Iron. *Adv. Mater*. **2020**, *32*, 1906910.

(4) Xu, J.; Cao, Z.; Zhang, Y.; Yuan, Z.; Lou, Z.; Xu, X.; Wang, X. A Review of Functionalized Carbon Nanotubes and Graphene for Heavy Metal Adsorption from Water: Preparation, Application, and Mechanism. *Chemosphere*. **2018**, *195*, 351-364.

(5) Lou, Z.; Cao, Z.; Xu, J.; Zhou, X.; Zhu, J.; Liu, X.; Ali Baig, S.; Zhou, J.; Xu, X. Enhanced Removal of As(III)/(V) From Water by Simultaneously Supported and Stabilized Fe-Mn Binary Oxide Nanohybrids. *Chem. Eng. J.* **2017**, *322*, 710-721.

(6) Huang, Y.; Zhang, W.; Zhang, M.; Zhang, X.; Zhao, Y. Hydroxyl-Functionalized TiO_2@SiO_2@Ni/nZVI Nanocomposites Fabrication, Characterization and Enhanced Simultaneous Visible Light Photocatalytic Oxidation and Adsorption of Arsenite. *Chem. Eng. J.* **2018**, *338*, 369-382.

(7) Gong, Y.; Gai, L.; Tang, J.; Fu, J.; Wang, Q.; Zeng, E.Y. Reduction of Cr(VI) in Simulated Groundwater by Fes-Coated Iron Magnetic Nanoparticles. *Sci. Total Environ.* **2017**, *595*, 743-751.

(8) Lv, D.; Zhou, J.; Cao, Z.; Xu, J.; Liu, Y.; Li, Y.; Yang, K.; Lou, Z.; Lou, L.; Xu, X. Mechanism and Influence Factors of Chromium(VI) Removal by Sulfide-Modified Nanoscale Zerovalent Iron. *Chemosphere*. **2019**, *224*, 306-315.

(9) Su, Y.; Adeleye, A.S.; Keller, A.A.; Huang, Y.; Dai, C.; Zhou, X.; Zhang, Y. Magnetic Sulfide-Modified Nanoscale Zerovalent Iron (S-nZVI) for Dissolved Metal Ion Removal. *Water Res.* **2015**, *74*, 47-57.

(10) Wu, D.; Peng, S.; Yan, K.; Shao, B.; Feng, Y.; Zhang, Y. Enhanced As(III) Sequestration Using Sulfide-Modified Nano-Scale Zero-Valent Iron with a Characteristic Core-Shell Structure: Sulfidation and as Distribution. *ACS Sustain. Chem. Eng.* **2018**, *6*, 3039-3048.

(11) Xu, J.; Avellan, A.; Li, H.; Clark, E.A.; Henkelman, G.; Kaegi, R.; Lowry, G.V. Iron and Sulfur Precursors Affect Crystalline Structure, Speciation, and Reactivity of Sulfidized Nanoscale Zerovalent Iron. *Environ. Sci. Technol.* **2020**, *54*, 13294-13303.

(12) Cao, Z.; Li, H.; Zhang, S.; Hu, Y.; Xu, J.; Xu, X. Properties and Reactivity of Sulfidized Nanoscale Zero-Valent Iron Prepared with Different Borohydride Amounts. *Environ. Sci. Nano* **2021**, *8*, 2607-2617.

(13) Meng, F.; Xu, J.; Dai, H.; Yu, Y.; Lin, D. Even Incorporation of Nitrogen into Fe^0 Nanoparticles as Crystalline Fe_4N for Efficient and Selective Trichloroethylene Degradation. *Environ. Sci. Technol.* **2022**, *56*, 4489-4497.
